# Nucleo-cytoplasmic distribution of SAP18 reveals its dual function in splicing regulation and heat-stress response in *Arabidopsis*

**DOI:** 10.1016/j.xplc.2024.101180

**Published:** 2024-10-31

**Authors:** Alvaro Santiago Larran, Jingyu Ge, Guiomar Martín, Juan Carlos De la Concepción, Yasin Dagdas, Julia Irene Qüesta

**Affiliations:** 1 Centre for Research in Agricultural Genomics (CRAG), CSIC-IRTA-UAB-UB, Campus UAB, Bellaterra, 08193 Barcelona, Spain; 2 Department of Biology, Healthcare and the Environment, Faculty of Pharmacy and Food Sciences, University of Barcelona, 08028 Barcelona, Spain; 3 Gregor Mendel Institute, Austrian Academy of Sciences, Vienna BioCenter, 1030 Vienna, Austria

**Keywords:** ASAP complex, plant development, alternative splicing, heat stress, *Arabidopsis thaliana*, biomolecular condensates, leaf development, thermoprotection, stress granules, shuttling proteins

## Abstract

Dynamic shuttling of proteins between the nucleus and cytoplasm orchestrates vital functions in eukaryotes. Here, we reveal the multifaceted functions of *Arabidopsis* Sin3-associated protein 18 kDa (SAP18) in the regulation of development and heat-stress tolerance. Proteomic analysis demonstrated that SAP18 is a core component of the nuclear apoptosis- and splicing-associated protein (ASAP) complex in *Arabidopsis*, contributing to the precise splicing of genes associated with leaf development. Genetic analysis further confirmed the critical role of SAP18 in different developmental processes as part of the ASAP complex, including leaf morphogenesis and flowering time. Interestingly, upon heat shock, SAP18 translocates from the nucleus to cytoplasmic stress granules and processing bodies. The heat-sensitive phenotype of a *SAP18* loss-of-function mutant revealed a novel role for SAP18 in plant thermoprotection. These findings significantly expand our understanding of the relevance of SAP18 for plant growth, linking nuclear splicing with cytoplasmic stress responses and providing new perspectives for future exploration of plant thermotolerance mechanisms.

## Teaser

This study provides evidence that SAP18 contributes to the splicing of genes related to leaf-development as a core component of the nuclear ASAP complex in *Arabidopsis; however, upon heat stress, SAP18 relocates to stress granules and processing bodies, playing an active role in thermotolerance. These findings suggest a multifaceted function of SAP18 in coordinating development and stress responses in plants.*

## Introduction

Plant development hinges on critical post-transcriptional processes such as alternative splicing, a key player in generating proteome diversity and organism complexity in response to diverse stimuli (Chaudhary et al., 2019). Within the intricate spliceosome machinery, proteins such as heterogeneous nuclear ribonucleoproteins (hnRNPs) and serine/arginine-rich (SR) proteins orchestrate splicing regulation through dynamic RNA interactions (Pan et al., 2008; Lee and Rio, 2015; Zhang et al., 2017). In addition to hnRNPs and SRs, Sin3-associated protein of 18 kDa (SAP18) stands out for its dual roles in transcriptional regulation and splicing (Knoepfler and Eisenman, 1999; Singh et al., 2010).

In animals, SAP18 acts as a co-repressor within the Sin3–histone deacetylase complex (Sin3–HDAC), linking it to transcription factors to modulate gene expression (Zhang et al., 1997; Schwerk et al., 2003). SAP18 also participates in a protein complex named the apoptosis- and splicing-associated protein (ASAP) complex, alongside RNA-binding protein S1 (RNPS1) and apoptotic chromatin condensation inducer in the nucleus (ACINUS) (Schwerk et al., 2003). This versatile complex is mainly involved in RNA processing, becoming a substructural component of the exon-junction complex (EJC) (Schwerk et al., 2003; Tange et al., 2005). The presence of a ubiquitin-like β-grasp fold in SAP18 further confirms its potential for multiprotein interactions (Zhang et al., 1997; Schwerk et al., 2003; Tange et al., 2005; Kiel and Serrano, 2006; McCallum et al., 2006). Notably, the structural integrity of SAP18 seems to be crucial for ASAP complex formation (Singh et al., 2010). Both the ASAP and the PSAP complex (in which ACINUS is replaced by its homolog PININ) are believed to function at the nexus of histone modification, transcription, and alternative splicing in metazoans (Schwerk et al., 2003; Murachelli et al., 2012; Deka and Singh, 2017).

In *Arabidopsis*, the Sin3–HDAC includes a SIN3-like protein (SNL1–6), SAP18, SAP30, one HDA protein (HDA19, HDA9, HDA7, or HDA6), and MULTICOPY SUPPRESSOR OF IRA1 (MSI1; Liu et al., 2014), which is also a core component of Polycomb repressive complex 2 (PRC2) (Derkacheva et al., 2013; Mehdi et al., 2016; Ning et al., 2019). SAP18 co-purifies with different PRC2 accessory proteins as well as with HDA19 (Qüesta et al., 2016). In addition, SAP18 has been shown to associate with various transcription factors, including ethylene responsive factors 4/5 (ERF4/5), SUPPRESSOR OF OVEREXPRESSION OF CONSTANS 1 (SOC1), and AGAMOUS-LIKE 24 (AGL24), which are involved in reproductive development (Song and Galbraith, 2006; Hill et al., 2008; Liu et al., 2009).

All the *Arabidopsis* ASAP components SAP18, ACINUS, and SERINE/ARGININE-rich 45 (SR45, orthologous to RNPS1 in animals) have been shown to associate with VIVIPAROUS1/ABSCISIC ACID INSENSITIVE3-LIKE1 (VAL1), a transcription factor involved in transcriptional inactivation of the floral repressor *FLOWERING LOCUS C* (*FLC*) leading to plant flowering (Qüesta et al., 2016; Mikulski et al., 2022). SR45 is crucial for plant splicing, as evidenced by defects in both constitutive and alternative splicing in *sr45* mutants (Zhang and Mount, 2009). SR45 binds exonic splicing enhancers, affecting splice-site selection and engaging early in spliceosome assembly (Day et al., 2012). In addition, SR45 governs alternative splicing of circadian clock genes to ensure rhythmic expression (Sanchez et al., 2010) and regulates other transcripts through motif-specific elements (Zhang et al., 2017). Furthermore, ACINUS is an integral component of U2/U12 spliceosomes and crucially interacts with SR45, influencing splicing activity (Palusa et al., 2007) and modulating abscisic acid (ABA) sensitivity by affecting the splicing of *ABA HYPERSENSITIVE 1* (*ABH1*) and *HYPERSENSITIVE TO ABA1* (*HAB1*) RNA (Bi et al., 2021).

Sequence analysis has predicted the formation of the ASAP complex in plants (Chen et al., 2019), and interactions between its three protein components have been anticipated (Chen et al., 2019; Bi et al., 2021). Moreover, the nuclear accumulation of SAP18-GFP in *Arabidopsis thaliana roots* is drastically reduced in the absence of SR45, indicating that SR45 may have a role in the maintenance of nuclear SAP18 protein levels (Chen et al., 2019). Correct expression of the ASAP complex has indeed been postulated to regulate *FLC*-dependent flowering (Mikulski et al., 2022). However, plant ASAP remains to be genetically characterized, and we lack a unanimous model for the interactions among its components. In particular, the potential role of SAP18 in splicing regulation has not been investigated. Despite some hints on the requirement for SAP18 to overcome high-salt conditions (Song and Galbraith, 2006), its broader roles in *Arabidopsis* stress responses have not been assessed to date. This limited understanding motivated our investigation into the functional role of SAP18 in *Arabidopsis*.

In this work, we reveal deeper insights into the complexity of SAP18-mediated cellular processes in *A*. *thaliana*. We present novel data suggesting that, as part of ASAP, nuclear SAP18 is actively involved in the regulation of leaf development and may have an independent cytoplasmic function, likely relevant to *Arabidopsis* thermotolerance.

## Results

### The interactome of SAP18 reveals its strong association with splicing

**Figure 1:**
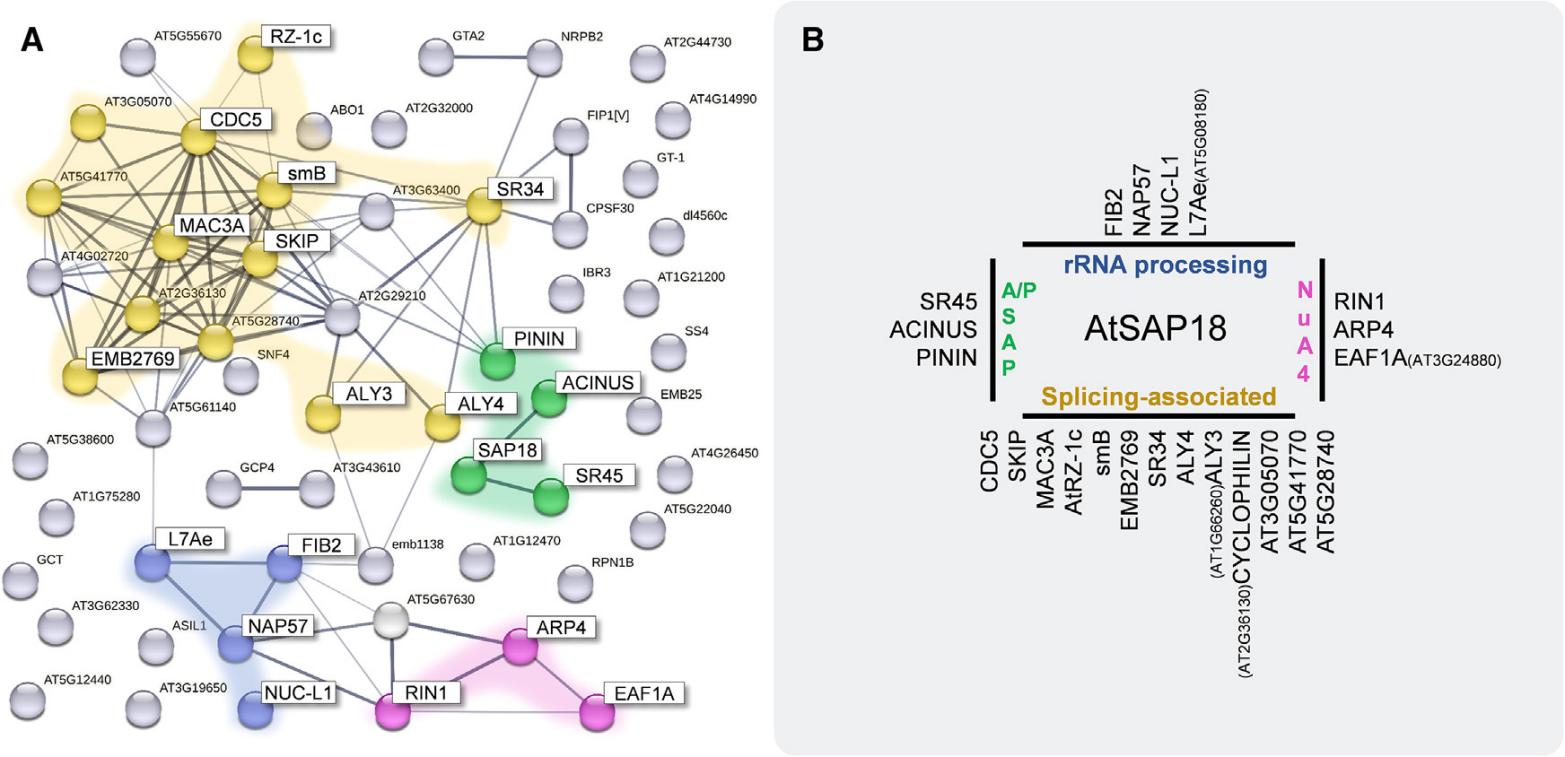
SAP18 interactome. **(A)** String network of SAP18-GFP interactors that resulted from the merging of two independent IP–MS experiments. Proteins in yellow correspond to components of the spliceosome or splicing-associated proteins, green to members of the ASAP/PSAP complex, blue to members of rRNA processing machinery, and pink to members of the NuA4 acetyltransferase complex. **(B)** Summary of the main complexes to which the interactors belong. The full list of interactors is provided in Supplemental Table 1.

To investigate the function of *Arabidopsis* SAP18, we performed immunoprecipitation followed by mass spectrometry (IP–MS) using plants overexpressing an SAP18-GFP fusion (provided by Chen et al., 2019) to identify SAP18 *in vivo* interactors. In all IP–MS experiments in this study, the *35S*:*GFP-*overexpression line was used as a control to eliminate non-specific hits. As a result of two independent experiments, each with three biological replicates, we were able to identify 58 high-confidence SAP18 interactors, which are summarized in a string network in Figure 1A. Line thickness indicates the strength of confidence for empirically validated interactions among nodes. The absence of lines between SAP18 and most of the nodes indicates that these interactions have not been reported or validated previously. In line with previous findings in animals, the largest group of SAP18 interactors are splicing-associated proteins (Figure 1B, yellow). Among these, we identified the ASAP/PSAP complex members ACINUS (AT4G39680), SR45 (AT1G16610), and AtPININ (AT1G15200) (Figure 1B, green) as top hits, confirming previously published data (Murachelli et al., 2012; Chen et al., 2019; Bi et al., 2021). In addition, SAP18 pulled out proteins involved in rRNA processing (such as homologs of NOPPL40-ASSOCIATED PROTEIN OF 57 kDa [NAP57] and FIBRILLARIN 2 [FIB2]) and proteins of the Nu4A acetyltransferase complex (Bieluszewski et al., 2015). The full list of candidate interactors can be found in Supplemental Table 1.

Despite previous reports suggesting involvement of the ASAP complex components SAP18 and SR45 in flowering through modulation of *FLC* levels via VAL1 (Mikulski et al., 2022), we were not able to recover VAL1 protein in our current settings, likely owing to differences in protein abundance. Likewise, neither HDA19 nor MSI1 (PRC2 complex component) arose as high-confidence interactors in our dataset, despite being described as SAP18 interactors previously (Mehdi et al., 2016; Qüesta et al., 2016). Our data clearly demonstrated that SAP18 is tightly linked to the splicing machinery and to ASAP complex components *in vivo* in seedlings. These findings prompted us to evaluate other poorly explored functions of SAP18 as a part of the ASAP complex in *Arabidopsis*.

### SAP18 takes part in the ASAP complex in the nucleus

**Figure 2:**
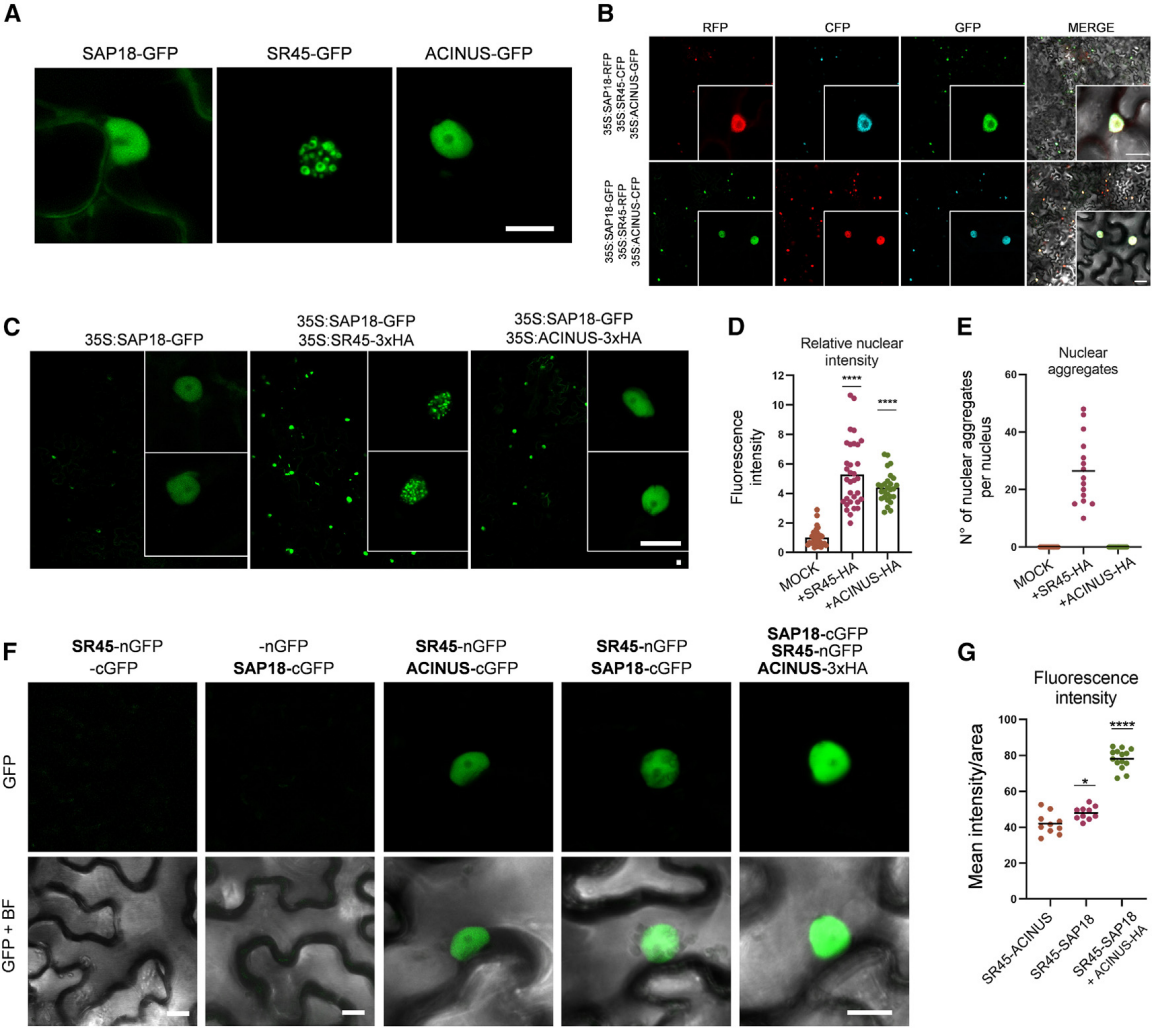
SAP18 subcellular localization in the context of the ASAP complex. **(A)** Representative images of SAP18-GFP, SR45-GFP, and ACINUS-GFP subcellular localization. **(B)** SAP18, SR45, and ACINUS localization in the nucleus of leaf epidermal cells of *N*. *benthamiana*. **(C)** Effect of SR45 and ACINUS on SAP18-GFP stabilization in leaf epidermal cells of *N*. *benthamiana*. **(D and E) (D)** Effect of SR45 and ACINUS on SAP18-GFP nuclear fluorescence intensity in 30 nuclei and **(E)** number of nuclear aggregates in leaf epidermal cells of *N*. *benthamiana* within 15 nuclei. **(F)** BiFC experiments using -nGFP and -cGFP fusions to evaluate interactions among SAP18, SR45, and ACINUS. **(G)** Mean fluorescence intensity/area of 10 images from BiFC experiments. Significance testing was performed by one-way ANOVA: ∗*p < 0.05* and ∗∗∗∗*p <* 0.0001. Scale bars in all microscopy images represent 10 μm.

Transient expression experiments in tobacco leaves confirmed that all ASAP proteins predominantly localize to the nucleus (Figure 2A). As previously reported, SR45 forms biomolecular condensates *in planta* (Figure 2A; Koroleva et al., 2009). The three members of the ASAP perfectly co-localize when co-expressed together, independently of the type of translational fusion used (Figure 2B). Unlike SR45 and ACINUS, SAP18 exhibited a unique subcellular distribution, being present in both the nucleus and the cytoplasm. This observation was supported by our SAP18 interactome data, which retrieved 7 out of 58 proteins (12.1%) that are predicted not to be nuclear (Supplemental Table 1).

Furthermore, our experiments revealed an intriguing dependence of SAP18 signal levels on the presence of SR45 and ACINUS. Upon co-expression with either SR45 or ACINUS, SAP18-GFP fluorescence in the nucleus became significantly brighter, evidencing the interaction between these components (Figure 2C and 2D). Notably, co-expression with SR45 led to formation of SAP18-GFP nuclear aggregates, whereas this did not happen upon co-expression with ACINUS (Figure 2E). Given that SR45 is known to form biomolecular condensates (Koroleva et al., 2009) and has recently been confirmed to undergo liquid–liquid phase separation (Zhang et al., 2022), these aggregates further support the association between SAP18 and SR45. We performed bimolecular fluorescence complementation (BiFC) experiments, which further confirmed the interaction between SAP18 and SR45 *in planta* (Figure 2F and Supplemental Figure 1). The SAP18–SR45 interaction was significantly enhanced in the presence of ACINUS (Figure 2F and 2G), likely as a result of stabilization of the *Arabidopsis* ASAP complex in the presence of all its three components.

To investigate the functional relevance of SAP18 nuclear retention by SR45, we performed additional IP–MS experiments using *Arabidopsis* plants overexpressing SAP18-GFP in the *sr45* mutant background (from Chen et al., 2019). Remarkably, none of the ASAP/PSAP components were detectable with confidence, and the proportion of non-nuclear interactors increased to 26 out of 82 (31.7%), providing further evidence that SAP18 function in the nucleus is dependent on the presence of SR45 (Supplemental Table 2). Therefore, although the subcellular localization of SAP18 is significantly shaped by its dynamic interactions with SR45 and ACINUS, possibly orchestrating vital cellular processes in the nucleus, our results also reveal a potential role for SAP18 in the cytoplasm that remains fully enigmatic.

### SAP18 contributes to regulation of *Arabidopsis* development by the ASAP complex

**Figure 3:**
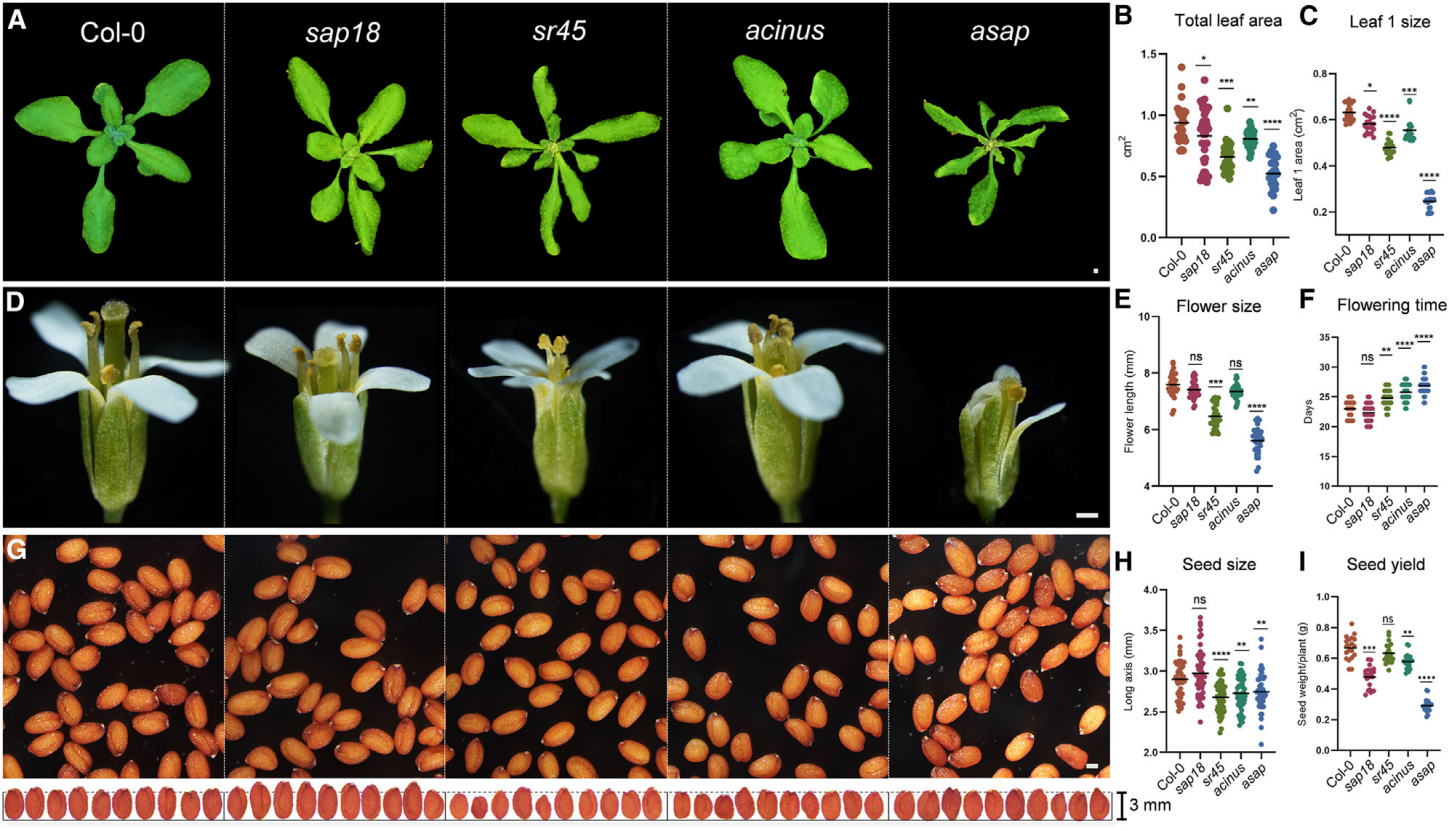
Development of *asap* mutants is severely affected. **(A–C) (A)** Rosette images of 25-day-old Col-0, *sap18*, *sr45*, *acinus*, and *asap* plants. Total leaf area **(B)** and leaf 1 size **(C)** was measured in 20 plants of each genotype and expressed in cm^2^. **(D)** Representative images of Col-0, *sap18*, *sr45*, *acinus*, and *asap* flowers. **(E)** Inflorescence length was measured in 30 flowers of each genotype and expressed in millimeters. **(F)** Flowering time was measured for 30 plants of each genotype growing at 22°C under a 16:8-h photoperiod. **(G)** Representative images of Col-0, *sap18*, *sr45*, *acinus*, and *asap* seeds. **(H)** The seed long axis was measured for 50 seeds of each genotype and expressed in millimeters. **(I)** Seed yield of Col-0, *sap18*, *sr45*, *acinus*, and *asap* genotypes was calculated as the average total seed weight produced per plant across 20 plants. Significance testing was performed by one-way ANOVA: ns, not significant (*p* > 0.05); ∗*p* < 0.05, ∗∗*p* < 0.01, ∗∗∗ *p* < 0.001, ∗∗∗∗*p* < 0.0001. Scale bars, 1 mm.

Although the contributions of SR45 and ACINUS to different aspects of *Arabidopsis* development and stress responses have been described individually (Ali et al., 2007; Zhang et al., 2017; Bi et al., 2021), the functions of both SAP18 and the plant ASAP complex as a whole continue to be poorly characterized. To start exploring the roles of ASAP in *Arabidopsis* development, we generated a mutant line carrying T-DNA insertions in the three ASAP components (hereafter referred to as the *asap* mutant) and compared the phenotypes of single (*sap18*, *sr45*, and *acinus*; Figure 3), double (*sap18 sr45*, *sap18 acinus*, and *sr45 acinus*; Supplemental Figure 2A), and triple mutant lines (*asap*; Figure 3). Overall, inactivation of ASAP complex components led to developmental defects during *Arabidopsis* vegetative and reproductive growth (Figure 3 and Supplemental Figure 2). Leaf development was compromised to some extent in all ASAP mutants (including single, double, and triple mutants), as reflected by the total leaf area of 25-day-old plants (Figure 3A–3C; Supplemental Figure 2A and 2B). *sap18* and *acinus* showed a slight reduction in total leaf area, a defect that was more evident in the *sr45* mutant and even more pronounced in the *asap* triple mutant (Figure 3B). *asap* plants also showed a significant reduction in leaf 1 area compared with each single mutant (Figure 3C). Remarkably, the equivalent total leaf area observed in *sap18 sr45* (Supplemental Figure 2B, 0.58 ± 0.14 cm^2^ compared with 0.93 ± 0.15 cm^2^ in Columbia-0 [Col-0]) and *asap* (Figure 3B, 0.52 ± 0.13 cm^2^ compared with 0.94 ± 0.16 cm^2^ in Col-0) suggested a prominent role of SAP18 and SR45 in the regulation of *Arabidopsis* leaf development.

Flowering time was delayed in *asap* mutants, likely owing to the combined effects of *sr45* and *acinus* (Figure 3F), as reported previously (Mikulski et al., 2022). In addition, flower development was greatly affected in *sr45*, and the defects were even greater in *asap* plants, in which defective flowers were also significantly reduced in size (Figure 3D and 3E). Moreover, we detected an abnormal number of petals in *sr45*, *acinus*, and *asap* mutants, with up to 45% of flowers exhibiting five petals in *acinus* and *asap* mutants (Supplemental Figure 2C and 2D). Seed size was also significantly affected in the *asap* mutant, as was the case for *sr45* and *acinus* single mutants (Figure 3G and 3H). However, seed yield and plant dry weight were substantially more affected in the *asap* mutant than in individual single mutants (Figure 3I; Supplemental Figure 2E and 2F).

Taken together, our observations confirm that the ASAP complex has pleiotropic roles in *Arabidopsis* development.

### ASAP complex prevents intron retention in genes associated with leaf development

**Figure 4:**
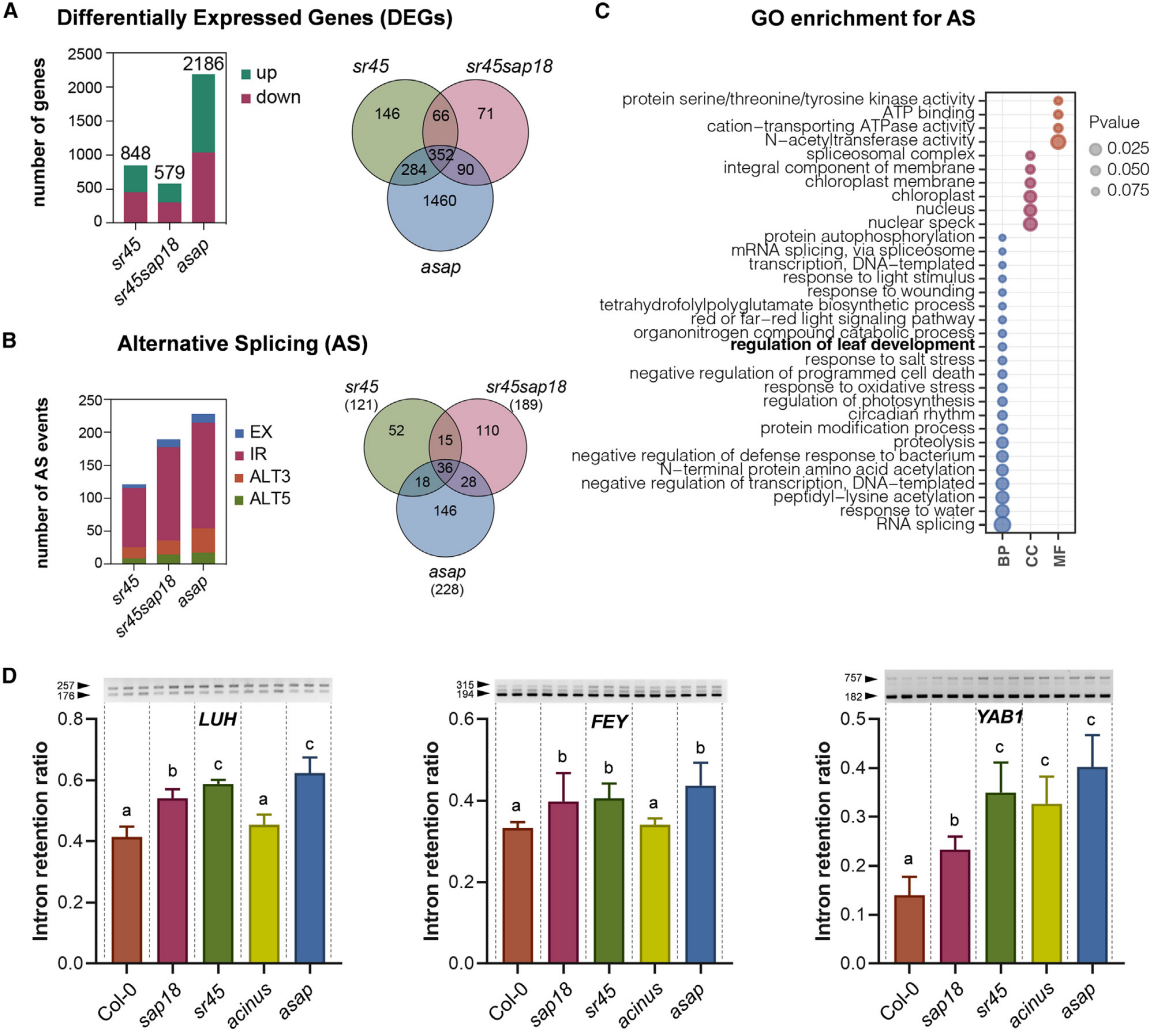
ASAP controls the splicing of *LUH*, *FEY*, and *YAB1* genes. **(A)** Venn diagram showing differentially expressed genes in *asap* compared with *sr45* and *sr45 sap18* mutants. **(B)** Venn diagram showing alternative splicing in *asap* compared with *sr45* and *sr45 sap18* mutants. **(C)** GO enrichment of biological process (BP), cellular compartment (CC), and molecular function (MF) terms for the whole set of alternatively spliced genes in all the mutants. **(D)** Quantification of intron-retained isoforms in *asap* mutants compared with Col-0, *sap18*, *sr45*, and *acinus*. DNA-free RNA samples isolated from 10-day-old seedlings were reverse transcribed, and variants were amplified by PCR. Products were separated on 2.5% agarose gels, and the intron-retention ratio was calculated as (upper band intensity)/(upper + lower band intensity) from triplicate reactions over two experiments. Details on primer locations and amplicon lengths are provided in Supplemental Figure 4A. Error bars show the standard deviation from the mean. Significance testing was performed by one-way ANOVA: “a,” “b,” and “c” indicate groups with statistically significant differences.

On the basis of the significant reduction in rosette size and leaf morphology phenotypes in the *asap* mutant (Figure 3A) and the strong genetic interaction observed in the *sr45 sap18* double mutant (Supplemental Figure 2A and 2B), we analyzed the transcriptomes of *sr45*, *sr45 sap18*, and *asap* mutants to examine possible cumulative effects on plant vegetative development. Gene-expression analysis revealed which genes were misregulated in each of the three mutants (Figure 4A and Supplemental Table 3). We observed a substantial overlap in the subsets of misregulated genes (Figure 4A) and in their expression patterns, which was expected given that SAP18 and SR45 form part of the ASAP complex (Supplemental Figure 3A). Gene ontology (GO) analysis of genes up- or downregulated in each of the analyzed mutants indicated an overlap in the biological processes regulated by SR45, SAP18, and the complete ASAP complex, although *asap* notably differed from the other genotypes in molecular functions (Supplemental Figure 3B). Moreover, the greater number of misregulated genes in the *asap* mutant (2186 vs. <1000 in the other genotypes) provided further evidence that additional functions of the ASAP complex require the full proficiency of all three protein components (Figure 4A).

Given the widely documented role of SR45 in splicing regulation (Zhang et al., 2017), we next analyzed alternative splicing profiles in the different mutants tested. In total, 121 alternative splicing events were detected in the *sr45* single mutant (Figure 4B and Supplemental Table 4). The majority of these events were retained introns, as expected in the absence of a functional splicing factor such as SR45. We also observed substantial overlap among the differential AS events in each mutant (Figure 4B and Supplemental Figure 3C). Accordingly, the aberrant proportion of retained introns was also observed in the *sr45sap18* and *asap* lines, indicating disfunction of the splicing process also in these mutants. The strongest magnitude of splicing changes occurred in the *asap* mutant, again confirming the cumulative effect of these mutations (Figure 4B). GO analysis revealed “regulation of leaf development” as one of the top biological processes affected by alternative splicing in the mutants (Figure 4C and Supplemental Table 4), consistent with the perturbed leaf developmental phenotypes, particularly of *asap* and *sap18 sr45* plants (Figure 3A and Supplemental Figure 2A).

We subsequently identified key regulators of leaf development that exhibited intron-retention events in the *asap* triple mutant: *FOREVER YOUNG* (*FEY*, AT4G27760), *FILAMENTOUS FLOWER* (*FIL*, also known as *YAB1*, AT2G45190), and *LEUNIG_HOMO- LOG* (*LUH*, AT2G32700) (Callos et al., 1994; Siegfried et al., 1999; Stahle et al., 2009) (Supplemental Figure 3C). To test the occurrence of these alternative splicing events, we quantified the abundance of intron-retained isoforms by RT–PCR, comparing Col-0 to *sap18*, *sr45*, *acinus*, and *asap* mutants. Our results indicated that intron-retained isoforms of *LUH*, *FEY*, and *YAB1* were more abundant in the *asap* mutant than in Col-0 (Figure 4D and Supplemental Figure 4A), likely because of a lack of functional SR45 protein. The increased intron retention in *FEY* and *YAB1* in all mutant genotypes was confirmed by RT–qPCR (Supplemental Figure 4B). Nevertheless, a significant intron-retention proportion was observed in the *sap18* single mutant compared with Col-0 for all three transcripts.

During the revision of this paper, we performed a new transcriptomic analysis of the *sap18* single mutant to investigate the individual contributions of SAP18. To account for potential batch effects, we included a new *sr45* sample as an internal control. Our analysis identified 183 differentially expressed genes (DEGs) in the *sap18* mutant compared with Col-0, versus 353 DEGs in the Col-0–*sr45* comparison, underscoring the more pronounced transcriptional misregulation associated with SR45 (Supplemental Figure 8A). Interestingly, we detected 140 AS events in *sap18* (Supplemental Figure 8B), although *FEY*, *LUH*, and *YAB1* were not among them (Supplemental Table 8). These findings highlight the importance of using high-sensitivity methods such as semi-quantitative RT–PCR for detection of low-abundance splice variants.

Together, our results confirm the major effect of SR45 on the ASAP splicing function, at the same time that they demonstrate a moderate contribution of SAP18 and ACINUS to this process.

### SAP18 subcellular localization upon heat shock: Beyond the nucleus

**Figure 5:**
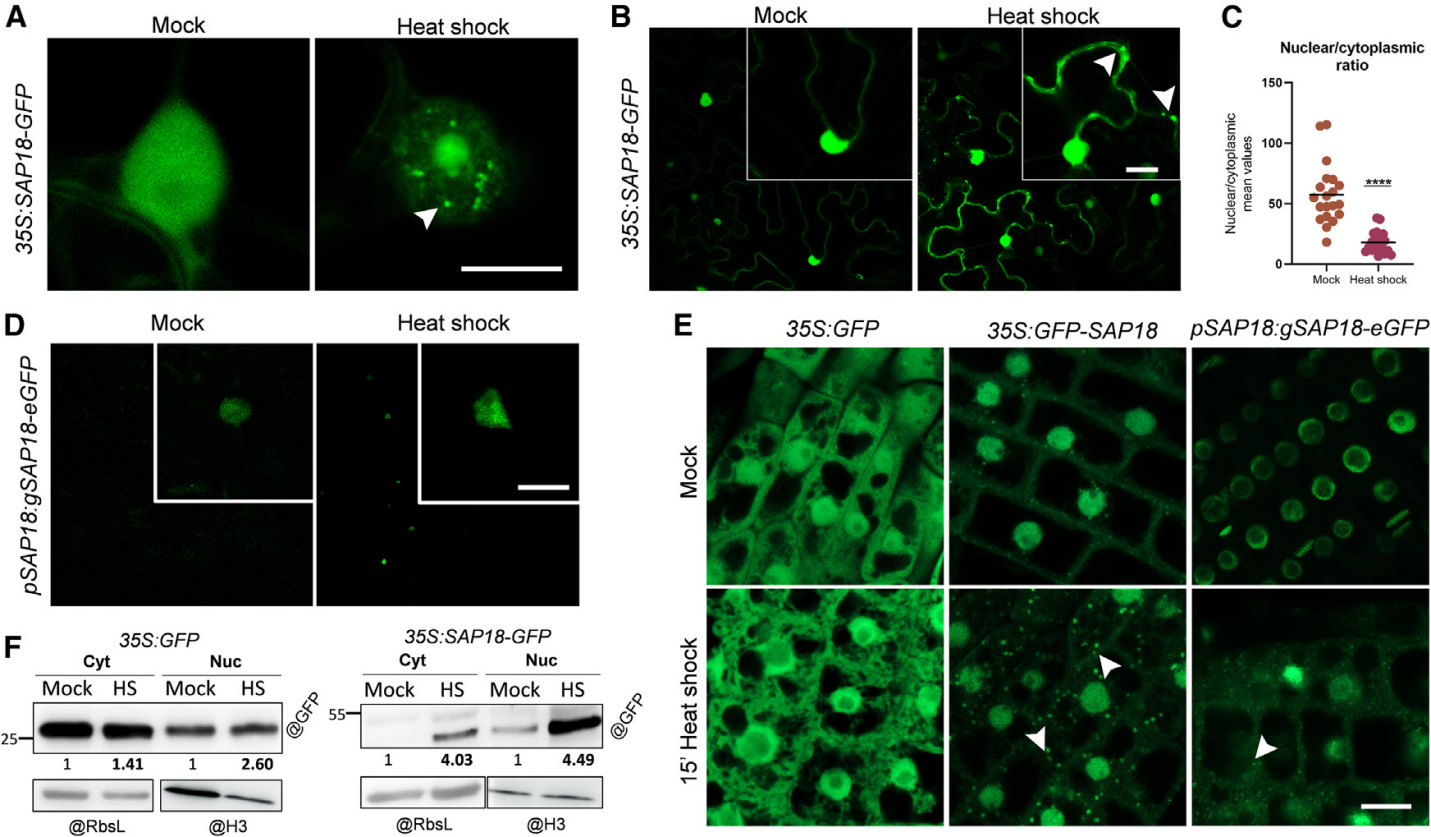
Subcellular localization of SAP18 upon heat shock. **(A)** Subnuclear localization of SAP18-GFP upon heat shock (HS) in epidermal cells of *N*. *benthamiana*. White arrows point to nuclear aggregates. **(B)** Effect of HS on the cytoplasmic localization of SAP18-GFP in epidermal cells of *N*. *benthamiana*. White arrows point to cytoplasmic aggregates. **(C)** Nuclear/cytoplasmic mean fluorescence intensity of SAP18-GFP in epidermal cells of *N*. *benthamiana* in mock vs. HS conditions (*n* = 20). **(D)** Effect of HS when SAP18 is expressed under the control of its endogenous promoter and terminator in *N*. *benthamiana*. **(E)** Effect of HS on SAP18-GFP localization in *A*. *thaliana* roots. White arrows point to cytoplasmic aggregates. **(F)** SAP18-GFP partitioning under HS. Nucleo-cytoplasmic fractionation and protein extraction were carried out using 1 g of *35S*:*GFP-* and *35S*:*SAP18-GFP*-overexpressing seedlings under mock conditions (22°C) or under HS (42°C for 2 h). Scale bars in all microscopy images represent 10 μm. Significance testing was performed with two-sided Student’s *t*-test: ∗∗∗∗*p* < 0.0001.

We next explored the dynamics of SAP18 localization under different stressors and found that the subnuclear localization of SAP18-GFP specifically changed upon heat shock (HS) (42°C for 2 h) but not under other stresses (Supplemental Figure 5B). SAP18-GFP displayed a strong tendency to form nuclear aggregates under HS (Figure 5A). Although mainly localized to the nucleus, some SAP18-GFP also accumulated in the cytoplasm (Figure 5B). This finding aligns not only with its association with splicing-related machinery (Figures 1 and 3D) but also with the dual localization previously observed for *Arabidopsis* SAP18 (Chen et al., 2019; Fanara et al., 2024) and its mammalian ortholog (Tange et al., 2005). Remarkably, *Nicotiana benthamiana* plants infiltrated with SAP18-GFP and exposed to HS exhibited an increase in cytoplasmic signal, as well as very discrete punctua that resembled biomolecular condensates in this compartment (Figure 5B and 5C; Supplemental Figure 5A; Supplemental Video 1).

Supplemental Video 1. Effect of heat shock on the cytoplasmic localization of SAP18-GFP in epidermal cells of *N. benthamiana*

To exclude the possibility that these effects were a product of overexpression, we built an SAP18 genomic construct (*pSAP18*:*gSAP18-eGFP-tSAP18*, hereafter pSAP18 construct) and repeated the HS experiments. As before, the GFP signal increased when *N*. *benthamiana* plants infiltrated with pSAP18 were subjected to HS treatment, with a clear effect of aggregate formation in the nucleus (Figure 5D).

Subsequently, we tested the effect of HS in *Arabidopsis* seedlings carrying the SAP18-GFP constructs. Formation of SAP18-GFP cytoplasmic aggregates occurred specifically in root cells of the *35S*:*SAP18-GFP* line and not in the *35S*:*GFP* control (Figure 5E). The appearance of cytoplasmic aggregates after HS was also evidenced in both roots (Figure 5E; full root tip in mock conditions can be found in Supplemental Figure 5C) and leaves (Supplemental Figure 5D) of the *Arabidopsis* line harboring the pSAP18 construct. Western blot analysis finally confirmed the nuclear and cytoplasmic partitioning of SAP18-GFP in *A*. *thaliana* seedlings, also revealing significant increases in the abundance of SAP18-GFP protein in both compartments upon HS (Figure 5F). Our results showed no significant differences in *SAP18* mRNA levels upon HS (Supplemental Figure 8C), indicating that this effect on protein accumulation was post-transcriptionally driven.

SAP18 localization thus appeared to be dynamic and responsive to changes in environmental conditions, in this case specifically, high-temperature stress. This novel discovery prompted us to investigate previously uncharacterized roles of SAP18 in the cytoplasm.

### SAP18 relocates to stress granules and processing bodies under heat shock

**Figure 6:**
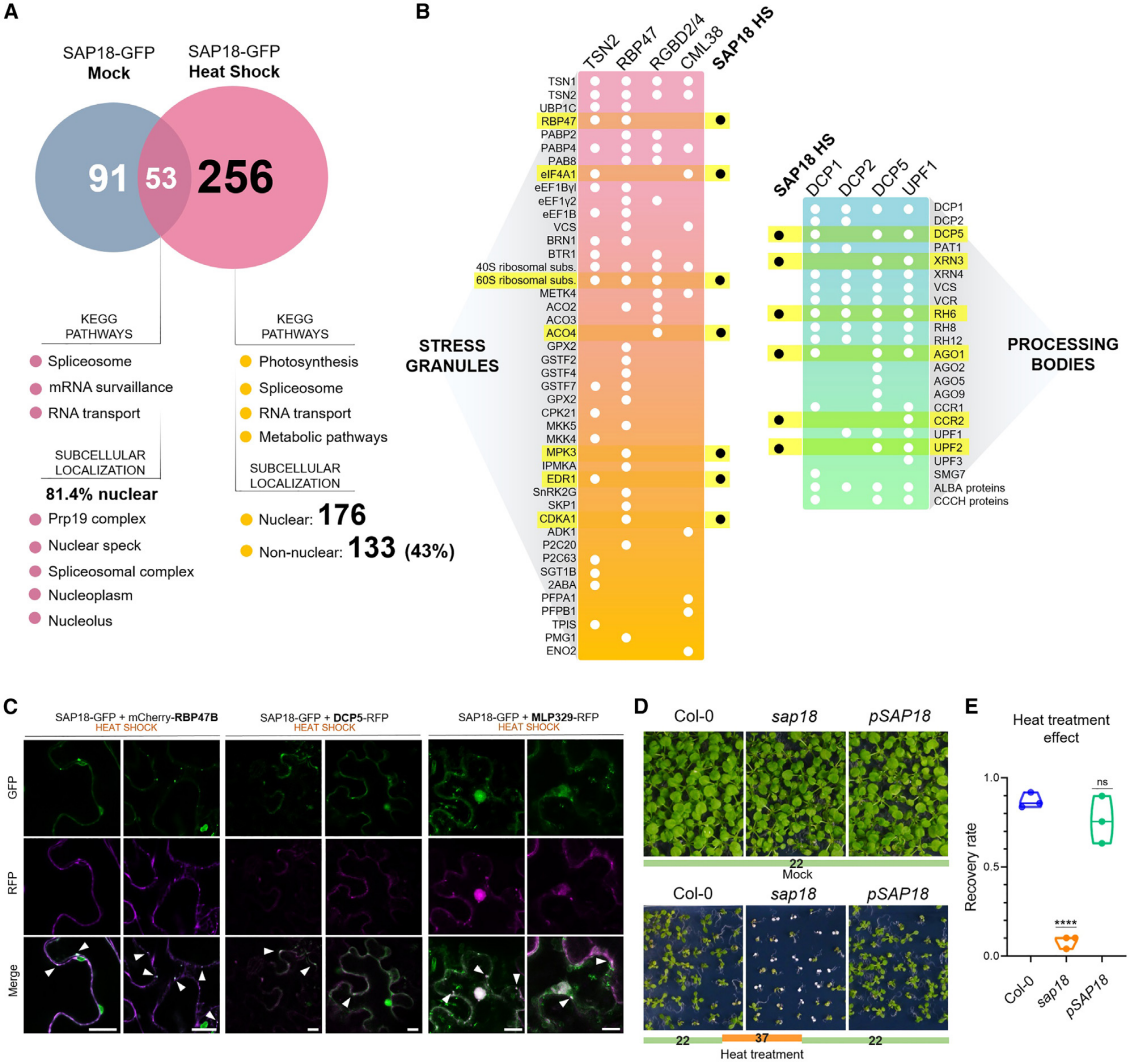
SAP18 co-localizes with stress granule markers upon HS. **(A)** Total number of SAP18 protein interactors among mock and HS conditions, with top categories of KEGG pathways and subcellular localization for SAP18 common interactors (both in mock and HS) and SAP18 HS-exclusive interactors. **(B)** SAP18 HS-exclusive interactors that have been reported to take part in cytoplasmic stress granules (SGs) and processing bodies (PBs) in previous datasets (Solis-Miranda et al., 2023). **(C)** Co-localization analysis in which SAP18-GFP was overexpressed together with RBP47B-mCherry (SG marker), DCP5 (PB marker), and MLP329-RFP in *N. benthamiana* plants subjected to HS. **(D)** Col-0, *sap18*, and pSAP18 seedlings subjected to HS treatment at 37°C for 4 days followed by a 10-day recovery at 22°C. Scale bar, 1 cm. **(E)** The recovery rate of each genotype is represented as the proportion of plants (50 plants, *n* = 3) that continued to produce green leaves after HS treatment. Significance testing was performed by one-way ANOVA: ∗∗∗∗*p* < 0.001; ns, not significant.

Given the observed changes in subcellular localization of SAP18 in response to HS, we decided to investigate whether the interactome changed in response to increased temperature. For this purpose, we performed additional SAP18-GFP IP–MS experiments after a 30-min treatment at 42°C. We observed substantial changes in the SAP18 interactome under HS conditions (Figure 6A), with a considerable increase in the number of interactors as well as a significant shift in the categories, biochemical pathways, and processes involved (Figure 6A and Supplemental Figure 6). There was a marked increase in the number of non-nuclear interactors (133/309, 43.0% compared with 12.1% under mock conditions, Supplemental Table 1), consistent with the higher abundance of SAP18-GFP in the cytoplasm under HS (Figure 5 and Supplemental Table 5). A significant number of these interactors were associated with photosynthesis, RNA transport, and metabolic pathways (Figure 6A and Supplemental Figure 6). Despite a large percentage of the interactome changing, 55 proteins remained as core interactors, including SR45, ACINUS, and PININ, most of which were primarily involved in cellular RNA processing and maintenance (Figure 6A). These observations further confirmed the strong association of SAP18 with the ASAP complex and its involvement in splicing regulation. The complete list of SAP18-GFP hits under HS is provided in Supplemental Table 3.

In line with the observed SAP18 cytoplasmic aggregates in response to heat, many of the SAP18 interactors identified under HS were proteins associated with stress granules (SGs) and processing bodies (PBs) (Figure 6B). For SGs, we identified core proteins related to mRNA biogenesis such as RBP47B, RBP47C, RBP45, translation initiation factors (eIF4, IF3-2), ribosomal subunit components (USI2Y), and protein kinases such as MDK8, EDR1, CDKA1, and CDKE-1. We also found numerous PB-associated proteins among the hits, including DCP5, XRN3, various RNA helicases (RH3, RH5, RH7, RH11, RH14, RH35, RH40, and RH50), AGO1, CCR1, and UFP2 (Solis-Miranda et al., 2023).

Given the large number of interactors exclusive to HS, we used AlphaFold multimer predictions to select proteins whose interaction likelihoods resembled those of the known SAP18 interactors ACINUS, PININ, and SR45. As shown in Supplemental Figure 7A, the components of the ASAP/PSAP complexes had the highest values of IPTM (Interface Predicted Template Modeling average score), a metric used to estimate the accuracy of a multimer model. Notably, among the other hits with similarly high IPTM values was MLP-LIKE PROTEIN 329 (MLP329), a protein previously reported to participate in *Arabidopsis* SGs (Kosmacz et al., 2019). We therefore tested the co-localization among SAP18, MLP329, RBP47B (a well-reported SG marker, Weber et al., 2008), and DCP5 (a well-reported PB marker; Xu and Chua, 2009) under HS. Our data demonstrated that SAP18 co-localizes with MLP329 in cytoplasmic aggregates upon HS in *N*. *benthamiana* (Figure 6C) but not under control conditions (Supplemental Figure 7B). In addition, SAP18 was included in RBP47B/TSN2 SGs (Figure 6C and Supplemental Figure 7B) and in DCP5 PBs (Figure 6C).

Together, our results indicate that SAP18 increases in abundance in response to HS in both the nucleus and the cytoplasm (Figure 5F), where it becomes part of an interface between SGs and PBs (Figure 6B and 6C). However, the specific function of SAP18 in these granules remains to be determined.

### SAP18 provides thermoprotection during heat stress

The aforementioned results led us to wonder whether SAP18 serves a functional role when plants experience heat stress. To answer this question, we tested whether SAP18 positively or negatively regulated plant thermotolerance. We performed a prolonged heat stress treatment by exposing 5-day-old Col-0 and *sap18* seedlings to 37°C for 4 days, after which the plants were recovered for 10 days at 22°C. All genotypes grew normally at 22°C under mock conditions (Figure 6D, left), but prolonged high-temperature treatment affected the growth of both plant genotypes. However, whereas the recovery rate of Col-0 plants was close to 1, growth of *sap18* mutant plants was severely impaired by the high-temperature treatment (Figure 6D [right] and 6E). The strong phenotype of *sap18* plants upon heat treatment was complemented by the SAP18 genomic construct, with pSAP18 lines exhibiting a recovery rate equivalent to that of wild-type (WT) Col-0 (Figure 6E). Likewise, the SAP18-overexpressing line behaved similarly to Col-0 plants after 37°C treatment (Figure 6E). Consequently, our results demonstrate that SAP18 protein is required for *Arabidopsis* thermotolerance.

## Discussion

### New perspectives on *Arabidopsis* SAP18 regulation

**Figure 7:**
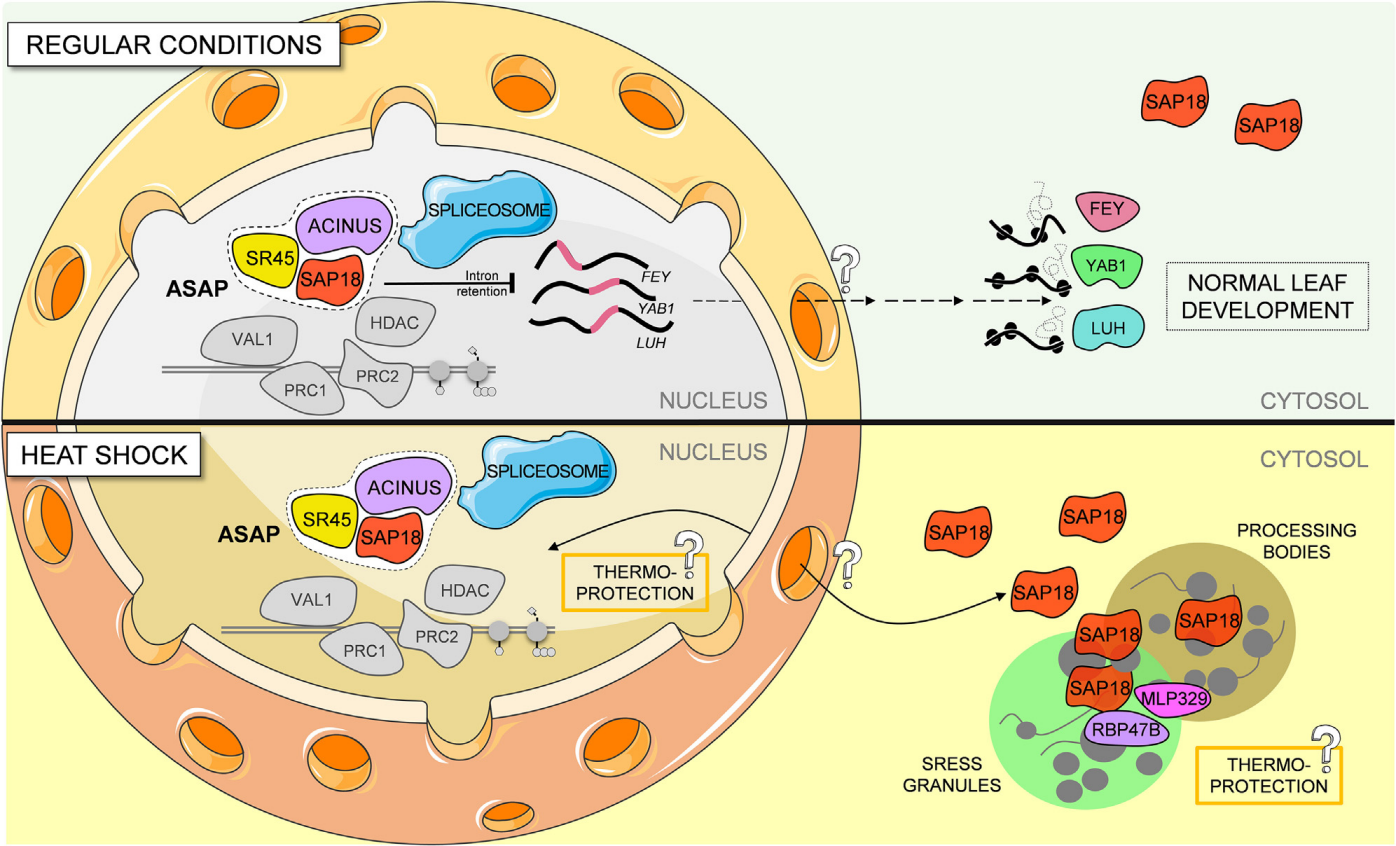
Proposed model for the action of SAP18 in *Arabidopsis thaliana.* SAP18 forms an integral part of the ASAP complex in the nucleus, which significantly impacts the precise splicing of genes associated with leaf development. Under HS, SAP18 relocates to stress granules and processing bodies in the cytoplasm, suggesting that its thermoprotective role may come from either its nuclear or cytoplasmic localization. Solid and dashed lines correspond to the movement of proteins and mRNAs, respectively. SAP18, SIN3-ASSOCIATED PROTEIN 18 KDA; SR45, SERINE/ARGININE-RICH 45; ACINUS, APOPTOTIC CHROMATIN CONDENSATION INDUCER IN THE NUCLEUS; FEY, FOREVER YOUNG; YAB1, FILAMENTOUS FLOWER (FIL); LUH, LEUNIG_HOMOLOG; RBP47B, RNA-BINDING PROTEIN 47B; MLP329, MLP-LIKE PROTEIN 329.

Our findings revealed multifaceted roles of SAP18 in *A*. *thaliana* (Figure 7). We showed that SAP18 forms an integral part of the ASAP complex, with a partial but significant impact on the splicing of genes associated with leaf development. Under HS, SAP18 relocates to SGs and PBs in the cytoplasm, allowing us to speculate that it may have a thermoprotective role under these conditions. These findings greatly enhance our understanding of SAP18’s versatile contributions to plant biology (Figure 7).

The identity of the high-confidence interactors revealed by affinity purification of SAP18-GFP followed by IP–MS under different experimental conditions prompted us to focus on aspects of *Arabidopsis* SAP18 regulation that were less studied (involvement in splicing regulation) or had not been described previously (cytoplasmic aggregates under HS). Nevertheless, SAP18 is well known for its interaction with histone deacetylation activity (Kumari et al., 2023) and its link to PRC silencing (Qüesta et al., 2016; Mikulski et al., 2022). In line with this, we detected peptides for HDA19 and MSI proteins in our datasets. However, these proteins did not pass the strict thresholds used in this work, including peptide count, fold change, and statistical significance across two independent experiments.

Other SAP18 interactors previously reported on the basis of *in vitro* assays, such as ERF4/ERF5 (Song and Galbraith, 2006), did not appear in our datasets. Similarly, whereas interactions with SOC1 and AGL24 have been validated *in vivo* through co-immunoprecipitation studies (Liu et al., 2009), these were performed in floral meristems, and SOC1 and AGL24 were thus less likely to be detected under our experimental conditions. In addition, we did not retrieve peptides for VAL1 protein, despite the fact that SAP18 was a top hit in IP–MS experiments that used VAL1 as bait (Qüesta et al., 2016; Mikulski et al., 2022). In this last case, we believe that protein abundance may explain the limitation in detecting VAL1. Spliceosome components, as well as the other members of the ASAP, SR45 and ACINUS, are highly abundant in all plant cells and may significantly outcompete less abundant proteins (such as a tissue-specific transcription factors like VAL1) in immunoprecipitation experiments. In summary, the IP–MS experiments performed in this work enabled us to explore novel features of the SAP18 protein, further reinforcing its important function in plant development and stress responses.

### Nuclear SAP18 contributes to the splicing of genes within the ASAP complex in *Arabidopsis*

In contrast to the well-established splicing roles of SR45 and ACINUS, *Arabidopsis* SAP18 has mainly been described as part of the Sin3-HDAC complex and, therefore, closely linked to epigenetic regulation of transcription (Song and Galbraith, 2006). Nevertheless, animal orthologs of SAP18 have been shown to regulate the splicing of both reporter and endogenous transcripts in an SR-domain-independent manner, likely via protein interactions mediated by its ubiquitin-like fold (Singh et al., 2010; Boehm et al., 2018).

Through the comprehensive analysis presented in this study, we revealed the role of SAP18 within the nucleus as a core component of the ASAP complex and splicing-associated proteins. Our transcriptomic data further support the function of the ASAP complex in alternative splicing. Intron-retention assays showed that SAP18 makes a moderate contribution to the proper splicing of *FEY*, *YAB1*, and *LUH*, all of which are associated with leaf development, an effect that was more evident in the *sr45* and *asap* mutants. Key findings from past studies indicate that *fey-1* mutants exhibit an abnormal number of cells in leaf primordia, accompanied by disruptions in leaf positioning and meristem maintenance (Callos et al., 1994). By contrast, *yabby1* mutants display narrow leaves and a partial loss of abaxial cell identity (Siegfried et al., 1999). Furthermore, *luh* homozygous mutants are embryonic lethal, whereas heterozygous mutants exhibit leaf-polarity defects (Stahle et al., 2009). LUH is a Gro/Tup1 co-repressor—like SAP18—and it interacts with the transcription factor YAB1, whereby they potentially collaborate in gene regulation and share common biological functions. Although none of the *luh*, *fey*, or *yabby1* single mutants had a leaf phenotype as strong as that of the *asap* mutant, multiple mutants do exhibit an enhanced phenotype. For instance, triple and quadruple mutants in the YAB family display a severe loss of abaxial cell identity with concomitant defects in leaf development (Stahle et al., 2009). One interesting possibility is that the cumulative effects on the reduction of functional isoforms of *YAB1*, *LUH*, and *FEY* transcripts due to open-reading-frame disruption (Supplemental Figure 4A) contribute to the *asap* phenotype. Intriguingly, the *sap18* single mutant exhibited significantly greater intron retention in all three *FEY*, *YAB1*, and *LUH* transcripts compared with WT plants (Figure 4D) without displaying the abnormal leaf-development phenotypes of the *sr45* and *asap* triple mutants. One possible explanation is that the proportion of intron-retained transcripts of *LUH* and *YAB1*, which was higher in *sr45* and *asap* than in *sap18*, could explain the leaf deformation. Alternatively, other misregulated genes could also be responsible for the abnormal leaf morphogenesis of *sr45* and *asap*.

Notably, *LUH*, *FEY*, and *YAB1* transcripts neither appeared misregulated nor showed differential RNA processing in the *sr45* mutant (Supplemental Tables 3 and 4). However, RNA molecules of the three genes were enriched in the RNA immunoprecipitation assay performed by Xing et al. (2015), suggesting that SR45 may serve as an anchor point for ASAP to regulate alternative splicing of the aforementioned leaf-development genes. Overall, our transcriptomic results point to the ASAP complex carrying out roles beyond SR45 and more dramatically affecting splicing.

On the other hand, members of the ASAP complex have been individually implicated in the regulation of different aspects of *Arabidopsis* development and stress responses. For instance, SR45 negatively regulates ABA signaling during early seedling development (Carvalho et al., 2010), and several abnormalities were reported for the *sr45* null mutant, including delayed root growth, smaller leaves, late flowering, narrower petals, shorter siliques with fewer seeds, and flowers with an unusual number of floral organs (Ali et al., 2007; Zhang et al., 2017). When ACINUS is defective, accumulation of unspliced U12 intron-containing transcripts occurs, leading to defects in ABA sensitivity (Bi et al., 2021). However, less information is available for SAP18 or for the whole complex in plants. By characterizing the *asap* mutant phenotype and its transcriptome, our study demonstrated the essential function of the ASAP complex in regulation of alternative splicing in plants. It would be intriguing to explore the outcome of a triple mutant that included *pinin* instead of *acinus*, or even a quadruple *sap18 sr45 acinus pinin* mutant. This would be especially relevant in light of the ASAP and PSAP complexes regulating the splicing of distinct subsets of transcripts and binding to the EJC core in a mutually exclusive manner (Wang et al., 2018). As for SAP18, our findings support the notion that it somehow affects splicing regulation, although this function could perhaps be exclusively attributed to its association with the ASAP complex. Future experiments, including RNA immunoprecipitation and a detailed motif analysis of the SAP18 structure, will help to determine whether SAP18 plays a specific role in alternative splicing in plants.

### SAP18 nuclear localization is promoted by SR45 and ACINUS

Although the ASAP was recognized as a stable trimeric complex belonging to the EJC, the interactions that provided stability to the complex were completely unknown until 2012, when mouse SAP18 was crystallized and some structural insights were revealed (Murachelli et al., 2012). SAP18 harbors a ubiquitin-like (UBL) fold domain at the C-terminus, and its N-terminus contains a conserved hydrophobic α/β groove-like structure suggested as essential for shaping the interaction surface between SAP18 and ASAP components. The UBL fold is crucial for the splicing role of SAP18 in animal models, as evidenced by the fact that a double Asp118 and Thr121 substitution in its core region impairs the splicing of a reporter RNA. This mutation also impairs the interaction with RNPS1 (SR45 animal homolog) and ACINUS, leading to the conclusion that the UBL domain is key for the integrity of the ASAP complex in animal models (McCallum et al., 2006; Singh et al., 2010). Supplemental Figure 7C shows a comprehensive organization of the *Arabidopsis* SAP18 domain structure based on conserved sequences and prediction tools. However, characterization of the SAP18 structure is still poor, and more structural insights are necessary to test functional domains.

Intriguingly, our data suggest a strong correlation between SAP18 nuclear localization and the presence of SR45 and ACINUS (Figure 2). This is also evidenced in our IP–MS dataset for the *sr45* mutant, in which the ASAP/PSAP complexes are notably absent (Supplemental Table 2). Nevertheless, the precise mechanism of physical interactions among these components remains unclear.

A prevailing model suggests that ACINUS acts as a linker within the ASAP complex, interacting with both SAP18 and SR45 through a conserved β hairpin called the RSB (RNPS1–SAP18-binding motif; Murachelli et al., 2012). Moreover, a direct interaction between SAP18 and SR45 has been reported (Bi et al., 2021). However, new investigations have cast doubt upon a direct interaction between ASAP complex members. Curiously, a recent report suggests that SAP18 and SR45 do not interact directly, requiring ACINUS as a key nexus for complex assembly (Wang et al., 2023); while another very recent paper proposed that SAP18 interacts with neither SR45 nor ACINUS alone and that the presence of all three proteins is necessary for the interaction to occur (Fanara et al., 2024). Using our experimental setup, we detected an interaction between SAP18 and SR45 through BiFC. This interaction significantly intensified when ACINUS was also present. Thus, our working hypothesis suggests a plant complex in which all proteins engage in physical interactions with each other and whose protein stability increases when all components are present simultaneously. Given the small size of SAP18 (18 kDa) and the fact that nuclear pores may typically allow the diffusion of proteins even larger than 60 kDa (Wang and Brattain, 2007), one interpretation could be that SAP18 may have the capacity to freely enter the nucleus and, once inside, may be retained by either SR45 or ACINUS. Moreover, our attempts at predicting nuclear localization signals within the SAP18 sequence were not successful, suggesting that there is no sequence element responsible for its nuclear localization. A comprehensive understanding of the interaction among members of the ASAP complex in plants will require further investigation, likely involving the resolution of its crystal structure to reveal key structural features.

### SAP18 dual localization and its role in *Arabidopsis* thermotolerance

In previous studies, SAP18 has been suggested to function as a shuttling protein. Initially, a heterokaryon analysis revealed that mouse SAP18 could localize in both the nucleus and the cytoplasm (Tange et al., 2005). Later, a proteomic study of SR45 revealed its influence on the nucleo-cytoplasmic distribution of SAP18 in *Arabidopsis* roots, revealing increased cytoplasmic abundance of SAP18 in the absence of SR45 (Chen et al., 2019). In addition, a recent publication also reported dual localization of SAP18 upon transient expression in tobacco leaves (Fanara et al., 2024). Nonetheless, none of these studies further explored the potential function of SAP18 in the cytoplasm.

Our observations revealed that SAP18 indeed exhibits a dual baseline localization, but this localization undergoes specific changes in response to high-temperature stress, both in transient expression systems and upon stable expression in *Arabidopsis* (Figure 5). In this context, we characterized changes in the SAP18 interactome under HS, which pointed at potential new functions that have remained uncharted until now.

In this work, we showed that SAP18 relocates to the cytoplasm after HS. The SAP18 interactome under HS conditions revealed the presence of numerous proteins associated with SGs and PBs, strongly implying that SAP18 may indeed be a constituent of these structures under such circumstances (Figure 6). We therefore performed co-localization experiments upon HS and observed that SAP18 translocated to cytoplasmic aggregates, co-localizing with RBP47B (an RNA-binding protein extensively associated with alternative splicing and stress responses in plants), DCP5 (a decapping protein required for PB formation), and MLP329, a protein whose functions are not fully understood but that is potentially involved in lipid binding within SGs (Kosmacz et al., 2019). Some of the hits from our HS dataset have been reported to behave similarly. For example, AGO1, a known component of PBs, has recently been described to associate with SGs under HS (Blagojevic et al., 2024).

These results led us to wonder whether SAP18 serves any functional role when plants experience heat stress. We observed that *sap18* mutants are thermosensitive, whereas pSAP18 complementation lines recovered to Col-0 levels after high-temperature treatment (Figure 6D and 6E). These findings hint at a novel, previously undisclosed thermoprotective role for *Arabidopsis* SAP18. In *Drosophila*, *sap18* mutants were demonstrated to exhibit thermosensitivity as well, and researchers postulated that dSAP18 potentially serves as a thermoprotective element during HS conditions (Costa et al., 2011). However, it remains to be determined whether nuclear or cytoplasmic SAP18 confers tolerance to high temperatures.

Because the conserved role of SAP18 has mainly been linked to mRNA splicing (Kumari et al., 2023), one possibility is that SAP18 mediates the production of specific splice variants under HS that contribute to plant thermotolerance. Indeed, splicing is important in plant HS responses (Rosenkranz et al., 2022). Although we still lack evidence of additional IR under HS for the *sap18* mutant (Supplemental Figure 8C), a thorough characterization of the *sap18* genome-wide spliceosome under high-temperature conditions could fully resolve whether nuclear SAP18 is required for thermotolerance. Another equally attractive possibility is that SAP18 regulation of thermotolerance may occur through a potential function within cytoplasmic SGs and PBs, as these types of cytoplasmic aggregates have been extensively linked to HS responses in plants (Hofmann et al., 2021). Further research will be required to fully clarify the involvement of SAP18 in *Arabidopsis* thermotolerance and determine whether it is due to its nuclear and/or cytoplasmic localization. This versatility of SAP18 offers exciting new directions to explore its role as a pivotal protein in plant adaptation to stressful environmental conditions.

## Methods

### Plant material and phenotype measurements

We used the *A. thaliana* Col-0 ecotype and *sap18* (SALK_083076), *sr45-1* (SALK_004132), and *acinus* (SALK_078554) single mutants. Plants were grown on Murashige and Skoog medium (MS medium) or soil substrate (as appropriate for each experiment) under a 16-h light (100 μmol m^−2^ s^−1^)/8-h dark regime (hereafter referred to as 16:8-h photoperiod) in a climate-controlled growth chamber (22°C). *asap* triple mutants were generated through crosses of *sap18 sr45* and *sap18 acinus* double mutants and propagated from plants homozygous for the three T-DNA insertions. *Arabidopsis* plants expressing *35S*:*SAP18-GFP* (in Col-0 and *sr45*) were provided by Prof. Xiao-Ning Zhang (Chen et al., 2019). Mutants were genotyped using the primers listed in Supplemental Table 6. Leaf phenotypes were recorded at specific developmental time points using a Nikon D-7000 camera. We calculated total leaf area and first leaf area using ImageJ, with a sample size of *n* = 20 plants per genotype. Reproductive phenotypes were documented by capturing images of flowers and siliques from 6-week-old plants using an Olympus SZX16 stereomicroscope. Flowering time was determined as the number of days post sowing when each plant developed a 2-cm-tall inflorescence stem, with 30 plants per genotype. Seed and inflorescence sizes were measured using ImageJ with sample sizes of *n* = 50 and *n* = 30, respectively. Seed yield per genotype was calculated by weighing the total seed production of 20 individually harvested plants per genotype. Aboveground biomass of each genotype was measured as the average dry weight of 20 plants after drying at 80°C for 72 h. For thermotolerance assays, *Arabidopsis* plants were grown on MS medium for 5 days at 22°C, shifted to 37°C for 4 days, and returned to 22°C for 10 days; recovery was then assessed and photographed using a Nikon D-7000 camera.

### Gene cloning and plant transformation

Coding sequences of *SAP18*, *SR45*, *ACINUS*, *MLP329*, and *DCP5* without stop codons were amplified using the primers listed in Supplemental Table 6 and cloned into the pENTR/D-TOPO vector (Invitrogen, Life Technologies, Carlsbad, CA, USA). These constructs were recombined using Gateway LR cloning into pGWB405 (35S:-GFP), pGWB454 (35S:-RFP), and pGWB644 (35S:-CFP) destination vectors for fluorescent protein fusions. Three genomic fragments (SAP18 coding region, 1440 bp from the ATG to the stop codon; SAP18 endogenous promoter, including the 5′ UTR, 1060 bp; 3′ UTR plus endogenous terminator, 880 bp) listed in Supplemental Table 7 were synthesized by Twist Bioscience and subcloned into a pBGW gateway binary vector using Golden Gate assembly. The resulting constructs were transformed into *Agrobacterium tumefaciens* GV3101 for transient and stable expression. Vectors were introduced into *Arabidopsis* via the floral dip method (Clough and Bent, 1998), and transgenic lines were selected in MS medium supplemented with basta/hygromycin/kanamycin before being propagated and selected to obtain homozygotes.

### Protein fractionation and western blotting

*Arabidopsis* plants expressing *35S*:*SAP18-GFP* and *35S*:*GFP* were grown for 10 days under a 16:8-h photoperiod at 22°C in MS medium. Approximately 1 g of seedlings was collected for either mock or HS treatment (42°C for 2 h) per duplicate. The tissue was ground in a mortar with liquid nitrogen, and 2 volumes of nuclei extraction buffer was added (100 mM MOPS [pH 7.6], 10 mM MgCl_2_, 0.25 M sucrose, 5% dextran T-40, 2.5% Ficoll 400, 1 tablet of protease inhibitor/10 ml, 3.12 μl/ml β-mercaptoethanol, 1 tablet of PhoStop/10 ml). The suspension was filtered through a double miracloth layer, and a 100-μl aliquot of the filtrate was collected for the total fraction. Following centrifugation at 10 000 *g* for 5 min at 4°C, the supernatant was retained as the cytoplasmic fraction. The pellet was resuspended in nuclei lysis buffer (50 mM Tris–HCl [pH 8], 10 mM EDTA [pH 8], 0.1% SDS) and sonicated three times for 10 s, yielding the nuclear fraction. Each fraction was supplemented with 2–4× sample buffer and boiled at 100°C for 10 min. Proteins were separated by SDS–PAGE and then transferred by western blotting to nitrocellulose membranes using a wet transfer blot system (Bio-Rad). Membranes were blocked with 5% skimmed milk in Tris-buffered saline and 0.1% Tween 20 (TBS-T) for 1 h at room temperature. Detection was performed using a rabbit polyclonal anti-GFP antibody (Abcam, #ab290, dilution 1:2500). Histone 3 (H3) and Ribosome Large subunit (RbsL) were used as nuclear and cytoplasmic markers, respectively, and detected using rabbit polyclonal anti-H3 (Agrisera, #AS10710, dilution 1:2500) and rabbit polyclonal anti-RbsL antibodies (Agrisera, #AS03037, dilution 1:2500). After five 5-min washes with TBS-T, blots were incubated with horseradish peroxidase-conjugated secondary anti-rabbit immunoglobulin G (GE, #NA934, dilution: 1:20 000). After five 5-min washes with TBS-T, the immune reaction was detected by chemiluminescence using Agrisera ECL SuperBright reagents and Amersham ImageQuant 800 western blot imaging systems. Protein band intensities were quantified using ImageJ. Equal rectangles were drawn around the bands of interest. The lane profile was obtained by subtracting the mean intensity of the background. The adjusted volume of the peak in the profile was taken as a measure of the band intensity. The protein band of interest was normalized for the total protein level of the whole lane.

### Immunoprecipitation and mass spectrometry

Plants expressing *35S*:*SAP18-GFP*(Col-0), *35S*:*SAP18-GFP*(*sr45*), and *35S*:*GFP*(Col-0) were grown for 10 days in MS medium under a 16:8-h photoperiod at 22°C. A subset of *35S*:*SAP18-GFP*(Col-0) and *35S*:*GFP*(Col-0) plants were subjected to HS (42°C for 2 h) before harvest. Approximately 1 g of plant material was harvested in triplicate for each genotype and treatment, ground in liquid nitrogen, and immediately dissolved in 2 volumes of extraction buffer (50 mM Tris–HCl [pH 7.5], 150 mM NaCl, 10 mM EDTA, 10% glycerol A2696 PanReac, 0.5% IGEPAL CA-630 I8896 Sigma, 0.5 mM dithiothreitol, 1 Roche protease inhibitor tablet/50 ml buffer, 1 g PVPP/50 ml buffer, 1 PhosSTOP tablet/10 ml buffer) by vortexing. Plant lysates were cleared by centrifugation at 16 000 *g* for 30 min at 4°C. The supernatant was filtered using 0.45-μm syringe filters and aliquoted into 1.5-ml LoBind tubes. This served as the input. An aliquot of 75 μl was reserved and mixed with 25 μl of 4× sample buffer. The mixture was heated at 100°C for 10 min and used as input. Anti-GFP magnetic beads (Chromotek) were equilibrated in extraction buffer for 1 h at room temperature. Thirty microliters of equilibrated beads per sample were used for immunoprecipitation, which was performed for 1 h at 4°C on a rotary shaker. The immunoprecipitated beads were collected in 1-ml aliquots in 1.5-ml LoBind tubes on a magnetic rack and washed twice with 1 ml of extraction buffer, followed by four washes with 1 ml of extraction buffer without IGEPAL. After excess liquid was discarded, 200 μl of extraction buffer was added to the beads; 20 μl of the bead suspension (equivalent to 10% of the total volume) was mixed with 20 μl of 2× sample buffer and incubated at 100°C for 10 min before undergoing SDS–PAGE and western-blotting protein checks. The remaining beads were stored at −20°C for subsequent MS analysis.

The total number of MS/MS fragmentation spectra was used to quantify each protein. For peptide identification, the RAW files were loaded into Proteome Discoverer (version 2.5.0.400, Thermo Scientific). All the resulting MS/MS spectra were searched using MS Amanda v.2.0.0.16129 (Dorfer et al., 2014). First, the RAW files were searched against the databases ID1140_SAP18.fasta (1 sequence; 152 residues), ID1173_eGFP.fasta (1 sequence; 239 residues), tags_v11.fasta (28 sequences; 2153 residues), TAIR10_pep_20101214_changeSymbol.fasta (32 785 sequences; 14 482 855 residues), and PD_Contaminants_TAGs_v20_tagsremoved.fasta. The peptide mass tolerance was set to ±10 ppm and the fragment mass tolerance to ±10 ppm. The maximal number of missed cleavages was set to 2. The result was filtered to 1% false discovery rate on protein level using the Percolator algorithm integrated in Thermo Proteome Discoverer. A sub-database was generated for further processing. Additional high-quality filtering was performed by setting a minimum MS Amanda score of 150 on peptide-spectrum match level. Protein areas were quantified using IMP-apQuant (Doblmann et al., 2019) by summing unique and razor peptides and using intensity-based absolute quantification (iBAQ; Schwanhäusser et al., 2011) with subsequent normalization based on the MaxLFQ algorithm (Cox et al., 2014). The pairwise comparisons were as follows: (1) SAP18-GFP in Col-0 vs. SAP18-GFP in *sr45*; (2) SAP18-GFP in Col-0 mock vs. SAP18-GFP in Col-0 under HS. In each case, comparisons were obtained from three independent biological replicates. Only proteins with log_2_(fold change) > 0, a *p* value of <0.05, and >3 peptides were considered for building Supplemental Tables 1, 2, and 5 and further analysis. Raw proteomics data are publicly available at the ProteomeXchange Consortium (Perez-Riverol et al., 2019) via the PRIDE partner repository with the dataset identifier PXD050985.

### Transient expression in *N*. *benthamiana* and BiFC

For transient expression, *N*. *benthamiana* plants were grown in controlled environmental conditions under a 16:8-h photoperiod at 22°C–25°C. *Agrobacterium* starter cultures expressing the constructs were prepared by inoculating a single colony in 2 ml of Luria broth with rifampicin, gentamycin, and spectinomycin selection and grown overnight at 28°C in a rotating shaker. Each starter culture was used to inoculate a working culture of 25 ml, which was grown overnight in the same medium and conditions. Working cultures were harvested by centrifugation at room temperature and 3000 *g* for 10 min. Cell pellets were resuspended in infiltration solution (10 mM MgCl_2_, 10 mM MES, pH 5.8) and the OD_600_ adjusted to 0.6 prior to addition of acetosyringone (200 μM). *Agrobacterium* suspensions were infiltrated using pressure on the abaxial surface of four leaves per plant with a disposable 5-ml syringe. In each experiment, at least two plants were infiltrated for each treatment/condition. Plants were returned to standard growth conditions for 48 h before imaging. For HS, a subset of infiltrated plants was exposed to 42°C for 2 h immediately before imaging.

SAP18, ACINUS, and SR45 were tested for BiFC by cloning into pB5nCGW, pB5cCGGW, pB5GWnC, pB5GWcCG, pB5nGGW, and pB5GWnG vectors (Kamigaki et al., 2016). Different combinations were co-infiltrated into *N*. *benthamiana* along with corresponding empty vector controls. Image acquisition and fluorescence intensity determination were performed as explained in the following section.

### Confocal microscopy and image processing

Transient expression in *N*. *benthamiana* was assessed by confocal microscopy imaging using a Leica SP5 system with a 20× objective lens, numerical aperture of 0.75, and laser excitation wavelengths of 488 nm (GFP), 561 nm (RFP), and 405 nm (CFP). A minimum of 20 frames were observed, and 10 frames were imaged per plant per treatment. Z-stack images were acquired with a step size of 1 μm. Confocal images were processed with ImageJ software (Schindelin et al., 2012). Fluorescence intensity was determined on images acquired using equal laser power, detector gain, and pinhole. A region of interest (ROI) was determined, and the mean fluorescence was measured. One ROI per frame was also selected in regions without fluorescence signal for background subtraction. Nuclear aggregate number (*n* = 15) and nuclear-to-cytoplasmic ratio (*n* = 20) were calculated when indicated in the figure legends. Statistical significance was determined using either two-sided Student’s *t*-test or one-way ANOVA with a threshold of *p* < 0.05.

For root imaging, 1-week-old *Arabidopsis* plants expressing *35S*:*GFP*, *35S*:*SAP18-GFP*, and *pSAP18*:*gSAP18-eGFP-tSAP18* were directly incubated at 37°C using a Zeiss 980 Elyra confocal microscope and imaged over time. Confocal imaging was performed using a 40× oil immersion objective with a numerical aperture of 1.4 and laser excitation at 488 nm. Z-projection images were generated from five optical sections with a step size of 1 μm. Observations were carried out on five roots per treatment condition.

### RNA extraction and sequencing

Col-0, *sr45*, *sap18 sr45*, and *asap* plants were grown on MS medium under a 16:8-h photoperiod at 22°C for 11 days. RNA was extracted from 100 mg of seedlings using an RNeasy plant mini kit (Qiagen) following the manufacturer’s instructions. RNA was quantified in a Qubit 2.0 fluorometer using a Qubit RNA Assay Kit followed by an integrity check on a Bioanalyzer. RNA (2 μg per sample) was sent to Macrogen for library preparation and sequencing (TruSeq stranded mRNA Library + NovaSeq 6000, 150 × 2 bp, 30M reads/sample, 4.5 Gb/sample).

### RNA-sequencing quantification of differential gene expression and alternative splicing

Total mRNA levels and inclusion of alternative sequences were quantified using *vast-tools* v.2.5 (Tapial et al., 2017). To identify DEGs between Col-0 and *sr45*, *sr45 sap18*, or *asap* mutants, we performed pairwise comparisons with the command *vast-tools_compare_expr*. The option -norm was added to our analysis to perform a quantile normalization of read counts between samples. In addition, genes with read counts <50 and that were not expressed at cRPKM >5 across all replicates of at least one of the two samples compared were filtered out. Finally, those genes with a fold change of at least 2 between each of the individual replicates from Col-0 and *sr45*, *sr45 sap18*, and *asap* mutants were respectively defined as SR45-, SR45SAP18-, or ASAP-regulated genes (Supplemental Table 3). During the revision of this paper, we performed a new transcriptomic analysis of the *sap18* single mutant, including a new *sr45* sample (*sr45*_new) as an internal control to account for potential batch effects.

To identify differentially spliced genes we mapped the RNA-sequencing (RNA-seq) data onto the araTha10 library, which is composed of an extended annotation of Ensembl Plants v.31 with all exon–exon and exon–intron junction sequences found in the *A*. *thaliana* genome (Martín et al., 2021). On the basis of this mapping, *vast-tools* provides the percentage of inclusion (PSI) of the putative alternative sequence using only exon–exon (or exon–intron for IR) junction reads and information on the read coverage that underlies the quantification of PSI (see https://github.com/vastgroup/vast-tools for details). The *vast-tools compare* command was then used to define differential alternative splicing events in each of the three mutants compared with Col-0 seedlings. To restrict our analysis and minimize the number of putative false positives, we performed these comparisons with the -min_ALT_use 25, -p_IR, and -noB3 *vast-tools compare* options (see https://github.com/vastgroup/vast-tools for details). Finally, splicing events with a |ΔPSI| > 20 between the compared pair of samples and whose PSI distribution did not overlap (-min_range 5) were defined as differential alternative splicing events (Supplemental Table 4).

### Gene ontology enrichment analyses of differentially expressed or spliced genes

To identify significantly enriched biological processes, molecular functions, or cellular components among the different sets of genes, analyses were performed using the functional annotation classification system DAVID (Huang et al., 2007).

### RT–qPCR

To measure SAP18 expression levels, plants were grown under control (22°C) and HS conditions (3 h at 37°C before harvesting) for 10 days, and 100 mg of tissue was sampled. RNA was extracted using an RNeasy Plant Mini Kit (Qiagen) following the manufacturer’s instructions. After reverse transcription using Maxima RT (Thermo Fisher Scientific), RT–qPCR was performed to detect relative gene expression levels using *ACTIN* as the internal reference gene. Each sample had three biological replicates, each with three technical replicates. Accumulation of the HS-inducible gene *HSFA7B* was measured as a positive control. Primers are listed in Supplemental Table 6.

### Intron-retention assay

Primers were designed to specifically amplify intron-retention variants of *LUH*, *FEY*, and *YABBY1* genes (Supplemental Table 6 and Supplemental Figure 4A). DNA-free RNA samples isolated from 10-day-old seedlings were reverse transcribed using Maxima RT (Thermo Fisher Scientific), and variants were amplified by PCR using the following conditions: initial denaturation at 95°C for 5 min; 30 cycles of denaturation at 95°C for 30 s, annealing at 53°C for 30 s, and extension at 72°C for 1 min/kb of the expected amplicon size; and a final extension step at 72°C for 3 min. PCR products were separated on 2.5% agarose gels. The intron-retention ratio was calculated as (upper band intensity)/(upper + lower band intensity) from triplicate reactions over 2–4 experiments. Intron retention was also assessed by RT–qPCR. In this case, we used two different primer pairs for each transcript: (1) the same primers used in the gel/PCR assays (correlating to the lower band in gel assays), which amplified the spliced version of the transcripts (spliced_var); and (2) a primer pair that specifically amplified the intron-retained variant of each transcript (IR_var). The intron-retention ratio was calculated as (IR_var)/(spliced_var + IR_var) from three biological replicates.

## Data and code availability

The MS proteomics data have been deposited at the ProteomeXchange Consortium via the PRIDE partner repository with the dataset identifier PXD050985. The RNA-seq datasets have been deposited in the NCIB Gene Expression Omnibus repository under the accession numbers GSE263007 and GSE280686. This paper does not report original code.

## Funding

This work was supported by the Junior Leader Fellowship [LCF/BQ/PI19/11690003] from “10.13039/100010434la Caixa” Foundation [ID100010434] and by grants from the 10.13039/501100004837Spanish Ministry of Science and Innovation (MCIN/AEI/10.13039/501100011033), including PID2019-110510GA-I00, EUR2021-122003, and CNS2023-145632 awarded to J.I.Q., PID2021-125223NA-I00 awarded to G.M., and the Severo Ochoa Excellence Programme for Centres (CEX2019-000902-S) awarded to CRAG. J.I.Q. (RYC2021-032539-I) and G.M. (RYC2020-030160-I) are Ramon y Cajal Fellows. Work at CRAG was also supported by the 10.13039/501100002809Generalitat de Catalunya (AGAUR, GRE2021, ref. SGR00873). Research in Y.D.’s lab is funded by the Austrian Academy of Sciences, Austrian Science Fund (FWF, P 34944), the 10.13039/501100002428Austrian Science Fund (FWF-SFB F79), the 10.13039/501100001821Vienna Science and Technology Fund (WWTF, LS21-009), and a European Research Council grant (project number: 101043370). A.S.L. received funding from the AGenT Programme, a European Union’s Horizon 2020 research and innovation Marie Skłodowska-Curie (MSCA) COFUND program under grant agreement no. 945043. J.G. is a recipient of a CSC fellowship funded by the 10.13039/501100004543China Scholarship Council. J.C.d.l.C. receives funding from the European Union’s Framework Programme for Research and Innovation Horizon 2020 (2014–2020) under the Marie Curie Skłodowska grant agreement no. 847548.

## Acknowledgments

We thank the facilities and services of CRAG and GMI for their support. We are grateful to Pawel Mikulski and Caroline Dean for providing *sap18*, *acinus*, and *sr45* mutant seeds and to Xiao-Ning Zhang for the *35S*:*gSAP18-GFP* seeds. We also thank Jose Julián Valenzuela for microscopy assistance at GMI. Finally, we thank Benjamin Tremblay and Melanie Ormancey for feedback on the manuscript.

## Author contributions

A.S.L. and J.I.Q. conceptualized the research project, designed the experiments, and interpreted the results. A.S.L. performed cloning, plant transformation, phenotypic analyses, BiFC, confocal microscopy, RNA-seq, and IP–MS experiments. A.S.L. wrote the manuscript with input from all authors. J.G. performed thermotolerance experiments. G.M. performed RNA-seq data analysis. J.C.d.l.C. provided guidance for IP–MS experiments and performed AlphaFold predictions. Y.D. provided funding and supervision for IP–MS experiments. J.I.Q. provided overall supervision and funding throughout the project’s execution and substantially participated in manuscript preparation and editing.

## Supplemental information

Document S1. Figures S1–S8

Supplemental Table 1. SAP18 interactors – mock

Supplemental Table 2. SAP18 interactors – *sr45* background

Supplemental Table 3. List of differentially expressed genes in *sr45*, *sr45sap18* and *asap* mutants

Supplemental Table 4. List of alternative splicing events detected in *sr45*, *sr45sap18* and *asap* mutants

Supplemental Table 5. SAP18 interactors – heat shock

Supplemental Table 6. Primers used in this study

Supplemental Table 7. DNA sequence of the fragments synthesized for cloning SAP18 genomic construct (pSAP18) Sequences highlighted in gray correspond to UTRs. Sequences in bold correspond to adaptors used for Golden Gate cloning. Underlined ATG corresponds to the SAP18 start codon.

Supplemental Table 8. List of differentially expressed genes and alternative splicing events detected in *sap18* mutant

Supplemental Data 1. IP–MS Experiment ID 1173 (SAP18-GFP in Col-0 *vs.* SAP18-GFP in *sr45* mutant)

Supplemental Data 2. IP–MS Experiment ID 1192 (SAP18-GFP mock *vs.* SAP18-GFP under Heat Shock)

Document S2. Article plus supplemental information
