# Supplementary material for: Nucleo-cytoplasmic distribution of SAP18 reveals its dual function in splicing regulation and heat-stress response in Arabidopsis
Source: Plant Commun. 2024 Oct 31;6(1):101180. doi: 10.1016/j.xplc.2024.101180 (PMC11784288; doi:10.1016/j.xplc.2024.101180)
Supplement: Document S1. Figures S1–S8 [file mmc1.pdf]

Plant Communications, Volume *6*

Supplemental information

Nucleo-cytoplasmic distribution of SAP18 reveals its dual function in splicing regulation and heat-stress response in *Arabidopsis*

Alvaro Santiago Larran, Jingyu Ge, Guiomar Martín, Juan Carlos De la Concepción, Yasin Dagdas, and Julia Irene Qüesta

SUPPLEMENTAL INFORMATION

Nucleo-cytoplasmic distribution of SAP18 reveals its dual function in splicing regulation and heat stress response in Arabidopsis

Alvaro Santiago Larran[1], Jingyu Ge[1], Guiomar Martín[1,2], Juan Carlos de la Concepción[3], Yasin Dagdas[3], Julia Irene Qüesta[1]

[1]Centre for Research in Agricultural Genomics (CRAG), CSIC-IRTA-UAB-UB, Campus UAB, Bellaterra, Barcelona, 08193, Spain.
[2]Department of Biology, Healthcare and the Environment, Faculty of Pharmacy and Food Sciences, University of Barcelona, Barcelona, 08028, Spain.
[3]Gregor Mendel Institute, Austrian Academy of Sciences, Vienna BioCenter, Vienna, 1030, Austria.

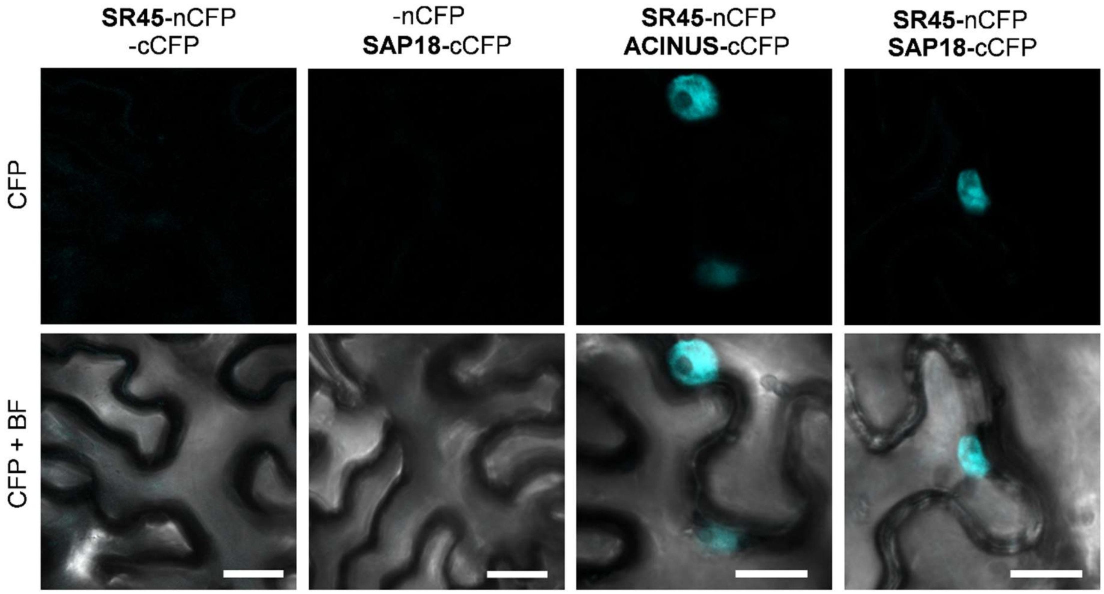

Supplementary Figure 1. BiFC experiments using -nCFP and –cCFP fusions to evaluate the interactions between SAP18, SR45 and ACINUS and support the interactions shown in Figure 2. Scale bar in all the microscopy images: 10 μm.

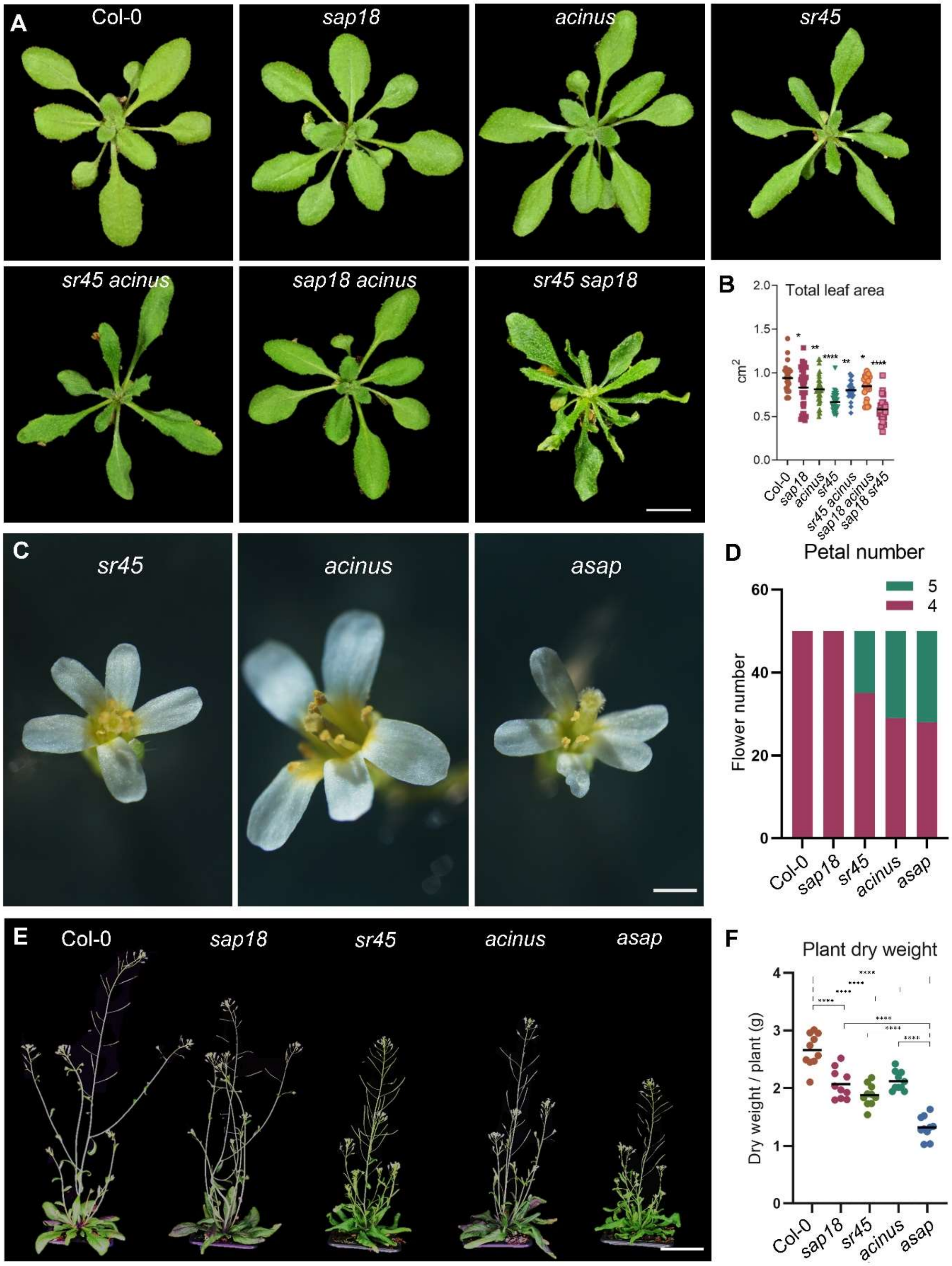

Supplementary Figure 2. (A) Top view of Col-0, *sap18*, *sr45*, *acinus*, *sr45 acinus*, *sap18 acinus* and *sr45 sap18* rosettes of 3-week old plants. (B) Total leaf area is represented as the average in total green area of 25 plants expressed in cm^2. Scale bar: 1 cm. (C) Top view of Col-0, *sap18*, *sr45*, *acinus* and *asap* flowers showing 5 petals. (D) Petal number distribution for each genotype is represented in the right graph. Scale bar: 1 mm. (E) Representative pictures of 50 day-old plants of Col-0, *sap18*, *sr45*, *acinus* and *asap*. (F) Average dry weight of Col-0, *sap18*, *sr45*, *acinus* and *asap* plants. Scare bar: 5 cm.

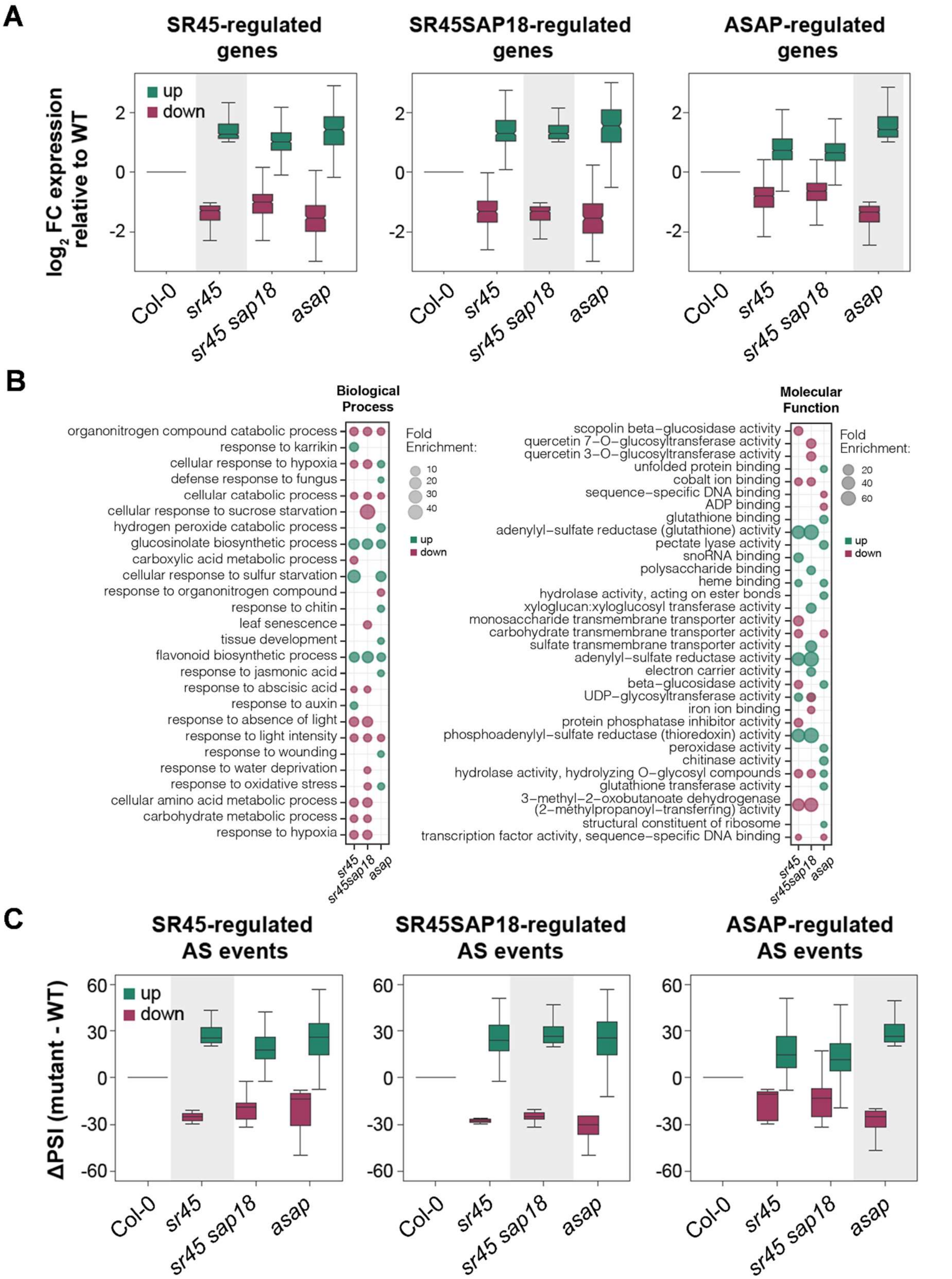

Supplementary Figure 3. (A) Log_2 fold-change expression of the up- and down-regulated genes in *sr45*, *sr45 sap18* and *asap* mutants compared to Col-0. (B) GO enrichment for differentially expressed genes (DEGs) regarding biological process and molecular function categories. (C) Differences in the Percentage of Spliced-In (PSI) versions for the genes alternatively spliced in *sr45*, *sr45 sap18* and *asap* mutants compared to Col-0.

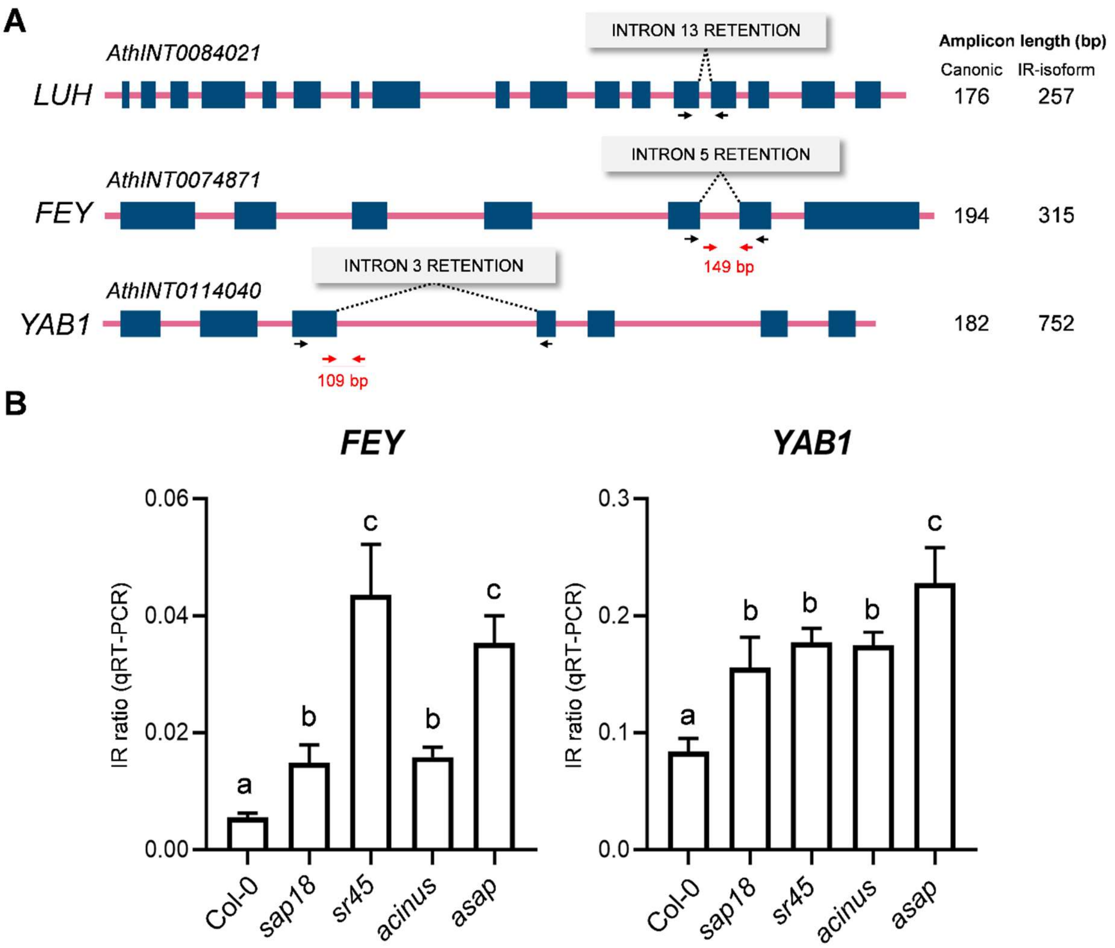

Supplementary Figure 4. (A) Intron retention events detected in *asap* mutants for the leaf development-associated genes *LUH, FEY* and *YAB1*. Black arrows represent the positions of primers used in RT-PCR assays of Figure 4D. (B) Intron Retention (IR) ratio of *FEY* and *YAB1* transcripts in Col-0, *sap18*, *sr45*, *acinus* and *asap* plants assessed by qRT-PCR. Two primer pairs depicted in (A) were used for each transcript. Black arrows indicate the primers used to amplify the spliced versions, while red arrows represent the primers used to amplify the intron-retained versions of each transcript. *ACTIN* was used as normalization control. Three biological replicates were analyzed per genotype. Significance testing was performed using one-way ANOVA.

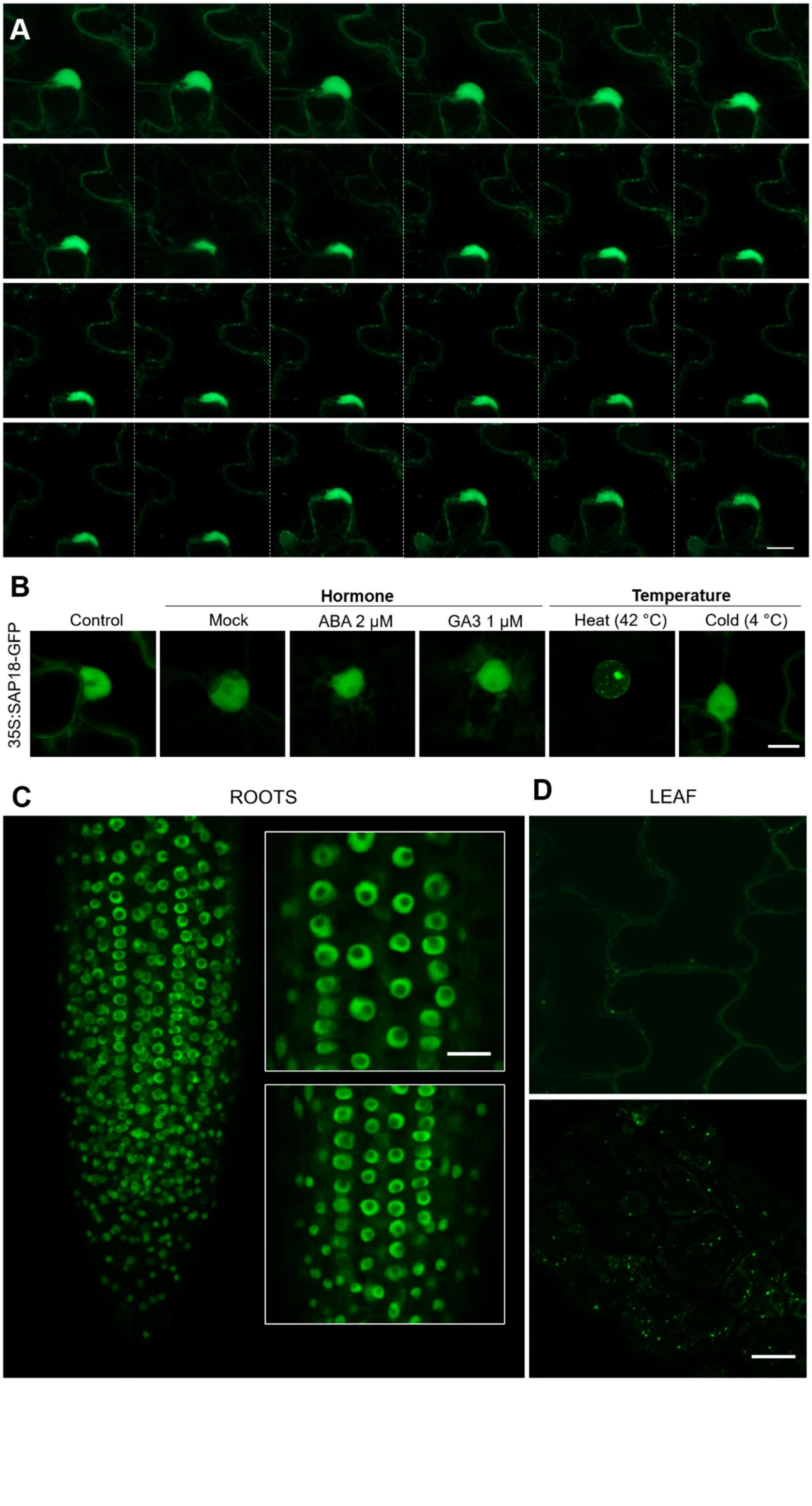

A
B
Hormone
Temperature
Control
Mock
ABA 2 μM
GA3 1 μM
Heat (42 °C)
Cold (4 °C)
35S:SAP18-GFP
C
ROOTS
D
LEAF

Supplementary Figure 5. (A) Time course of SAP18-GFP localization upon HS. Individual pictures of *N. benthamiana* leaf epidermis expressing SAP18-GFP taken every 2 minutes. Bar scale: 10 µm. (B) Representative pictures of SAP18 subcellular localization in *N. benthamiana* plants upon exposure to different treatments. Scale bar: 10 µm. SAP18 endogenous expression in roots (C) and leaves (D) of 7-day-old *A. thaliana* seedlings harbouring the construction pSAP18:gSAP18-eGFP-tSAP18. Scale bars: 10 µm.

A

KEGG Pathways

pathway	description	count in network	strength	false discovery rate
ath03040	Spliceosome	11 of 188	1.25	7.36e-09
ath03018	RNA degradation	6 of 110	1.22	0.00016
ath03015	mRNA surveillance pathway	5 of 114	1.13	0.0021

Molecular Function (Gene Ontology)

GO-term	description	count in network	strength	false discovery rate
GO:0043015	Gamma-tubulin binding	3 of 3	2.48	0.00024
GO:0034513	Box H/ACA snoRNA binding	2 of 4	2.18	0.0301
GO:0030515	snoRNA binding	3 of 14	1.81	0.0052
GO:0003724	RNA helicase activity	4 of 90	1.13	0.0422
GO:0004386	Helicase activity	5 of 156	0.99	0.0333
GO:0003729	mRNA binding	10 of 332	0.96	7.72e-05
GO:0003723	RNA binding	25 of 954	0.9	1.22e-12
GO:0005198	Structural molecule activity	10 of 412	0.87	0.00033
GO:0140098	Catalytic activity, acting on RNA	7 of 320	0.82	0.0213
GO:0003676	Nucleic acid binding	33 of 2854	0.55	4.42e-08
GO:1901363	Heterocyclic compound binding	41 of 5413	0.36	2.06e-05
GO:0097159	Organic cyclic compound binding	41 of 5434	0.36	2.06e-05
GO:0005488	Binding	50 of 8453	0.26	0.00032

B

KEGG Pathways

pathway	description	count in network	strength	false discovery rate
ath00196	Photosynthesis - antenna proteins	6 of 21	1.48	6.85e-06
ath00450	Selenocompound metabolism	3 of 18	1.25	0.0209
ath00195	Photosynthesis	12 of 74	1.24	1.79e-09
ath03040	Spliceosome	25 of 188	1.15	3.38e-18
ath03013	RNA transport	9 of 165	0.76	0.0013
ath00270	Cysteine and methionine metabolism	6 of 118	0.73	0.0209
ath01100	Metabolic pathways	39 of 2275	0.26	0.0056

Molecular Function (Gene Ontology)

GO-term	description	count in network	strength	false discovery rate
GO:0008705	Methionine synthase activity	3 of 3	2.03	0.0016
GO:0003871	5-methyltetrahydropteroyltriglutamate-homocysteine S-met...	3 of 3	2.03	0.0016
GO:0030628	pre-mRNA 3-splice site binding	3 of 7	1.66	0.0068
GO:0016168	Chlorophyll binding	10 of 30	1.55	7.99e-10
GO:0045156	Electron transporter, transferring electrons within the cyclic ...	3 of 11	1.46	0.0170
GO:0036002	pre-mRNA binding	4 of 16	1.43	0.0025
GO:0008143	poly(A) binding	3 of 17	1.27	0.0422
GO:0019904	Protein domain specific binding	8 of 59	1.16	3.26e-05
GO:0003724	RNA helicase activity	11 of 90	1.12	6.36e-07
GO:0003727	Single-stranded RNA binding	6 of 59	1.04	0.0025
GO:0003755	Peptidyl-prolyl cis-trans isomerase activity	5 of 54	0.99	0.0145
GO:0004386	Helicase activity	13 of 156	0.95	1.37e-06
GO:0003729	mRNA binding	25 of 332	0.9	2.41e-12
GO:0003723	RNA binding	54 of 954	0.78	1.42e-22
GO:0140098	Catalytic activity, acting on RNA	18 of 320	0.78	7.12e-07
GO:0046906	Tetrapyrrole binding	14 of 297	0.7	0.00017
GO:0003924	GTPase activity	7 of 156	0.68	0.0439
GO:0017111	Nucleoside-triphosphatase activity	22 of 666	0.55	7.40e-05
GO:0016462	Pyrophosphatase activity	23 of 709	0.54	6.28e-05
GO:0016887	ATPase activity	15 of 510	0.5	0.0091
GO:0003676	Nucleic acid binding	70 of 2854	0.42	1.85e-11
GO:0043168	Anion binding	55 of 2682	0.34	5.22e-06
GO:0097159	Organic cyclic compound binding	107 of 5434	0.32	1.22e-12
GO:1901363	Heterocyclic compound binding	106 of 5413	0.32	2.00e-12
GO:0046872	Metal ion binding	54 of 3023	0.28	0.00033
GO:0005488	Binding	146 of 8453	0.27	1.05e-14
GO:0043167	Ion binding	90 of 5185	0.27	2.81e-07
GO:0016787	Hydrolase activity	41 of 2363	0.27	0.0079
GO:0035639	Purine ribonucleoside triphosphate binding	37 of 2172	0.26	0.0208
GO:0097367	Carbohydrate derivative binding	38 of 2257	0.25	0.0209
GO:0032555	Purine ribonucleotide binding	37 of 2206	0.25	0.0263
GO:0003824	Catalytic activity	93 of 6726	0.17	0.0019

Supplementary Figure 6. (A) KEGG pathways and GO molecular Function for SAP18 interactors in mock conditions. (B) KEGG pathways and GO molecular Function for SAP18 interactors under heat shock. Tables were obtained using STRING (string-db.org).

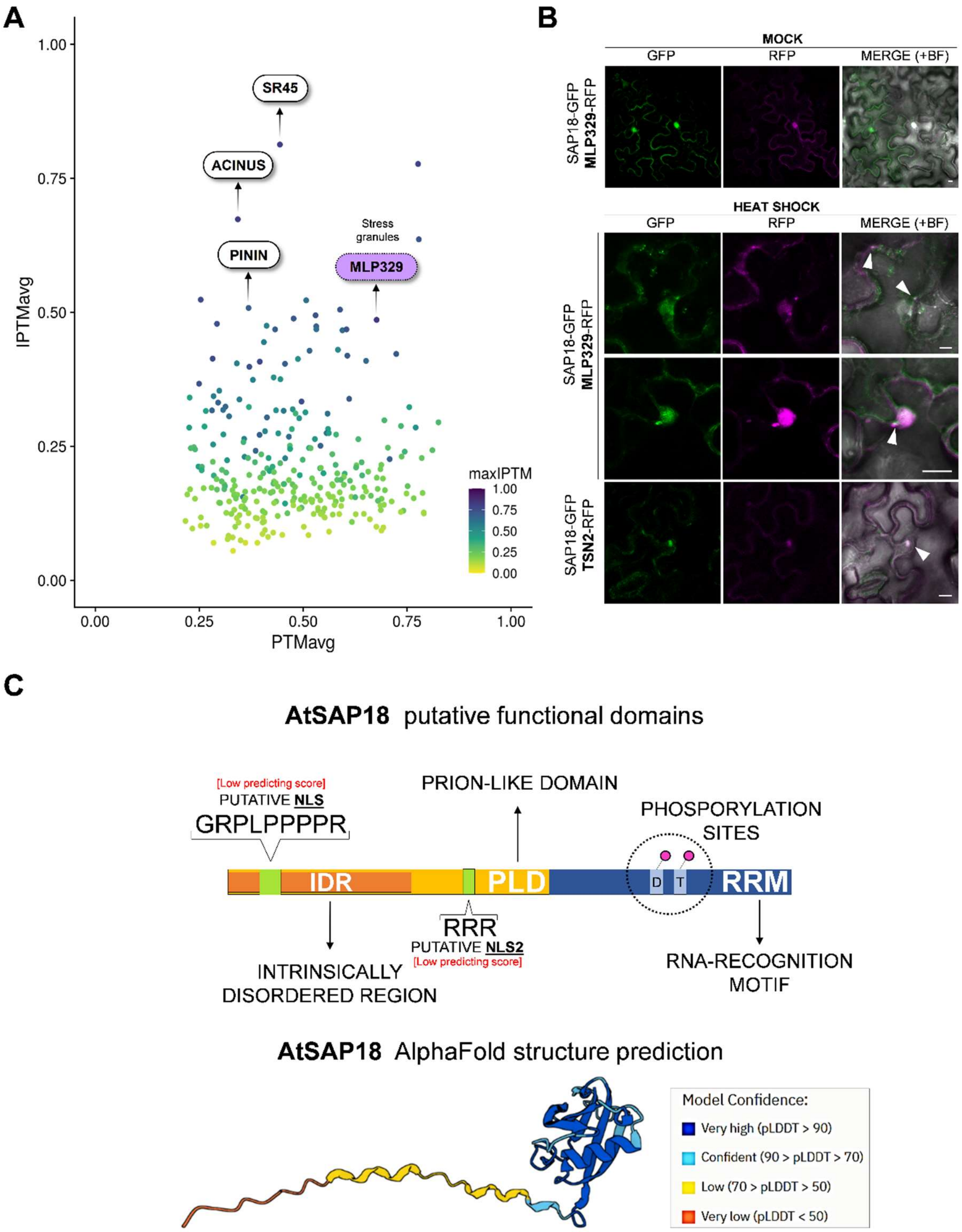
A
1.00
0.75
0.50
0.25
0.00
IPTMavg
0.00
0.25
0.50
0.75
1.00
PTMavg
SR45
ACINUS
PININ
Stress granules
MLP329
maxIPTM
1.00
0.75
0.50
0.25
0.00
B
MOCK
GFP
RFP
MERGE (+BF)
SAP18-GFP
MLP329-RFP
HEAT SHOCK
GFP
RFP
MERGE (+BF)
SAP18-GFP
MLP329-RFP
SAP18-GFP
TSN2-RFP
C
AtSAP18 putative functional domains
[Low predicting score]
PUTATIVE NLS
GRPLPPPPR
PRION-LIKE DOMAIN
PHOSPORYLATION SITES
IDR
PLD
D
T
RRM
RRR
PUTATIVE NLS2
[Low predicting score]
INTRINSICALLY DISORDERED REGION
RNA-RECOGNITION MOTIF
AtSAP18 AlphaFold structure prediction
Model Confidence:
Very high (pLDDT > 90)
Confident (90 > pLDDT > 70)
Low (70 > pLDDT > 50)
Very low (pLDDT < 50)

Supplementary Figure 7. (A) *In silico* prediction of the interaction between SAP18 and the full list of HS-exclusive interactors using AlphaFold-Multimer predictions. PTMavg (Predicted Template Modelling average score) and IPTMavg (Interface Predicted Template Modelling average score) are measures of the congruency of protein structures considering residues of the same or different chains, respectively. (B) Co-localization analysis overexpressing SAP18-GFP together with MLP329-RFP and TSN2-RFP using *Nicotiana benthamiana* plants subjected to HS. Scale bar: 20 µm. (C) SAP18 structural domain organization. We used AIUPred (https://iupred.elte.hu/) for the prediction of intrinsically unstructured protein regions, and SMART (Simple Modular Architecture Research Tool; http://smart.embl-heidelberg.de/help/smart_about.shtml) to explore domain architectures. NLStradamus online tool (http://www.moseslab.csb.utoronto.ca/NLStradamus/) with a cutoff value of 0.2 was used to predict the detection of any potential NLS. Annotations were curated using previously published data for SAP18 in animal models (McCallum et al., 2006; Murachelli et al., 2012), especially for point amino acids 131D and 134T which were predicted as phosphorylation sites with functional implications (Singh et al., 2010). NLS: Nuclear Localization Signal; PLD: Prion-Like Domain; RRM: RNA-Recognition Motif (which matches the ubiquitin-like fold).

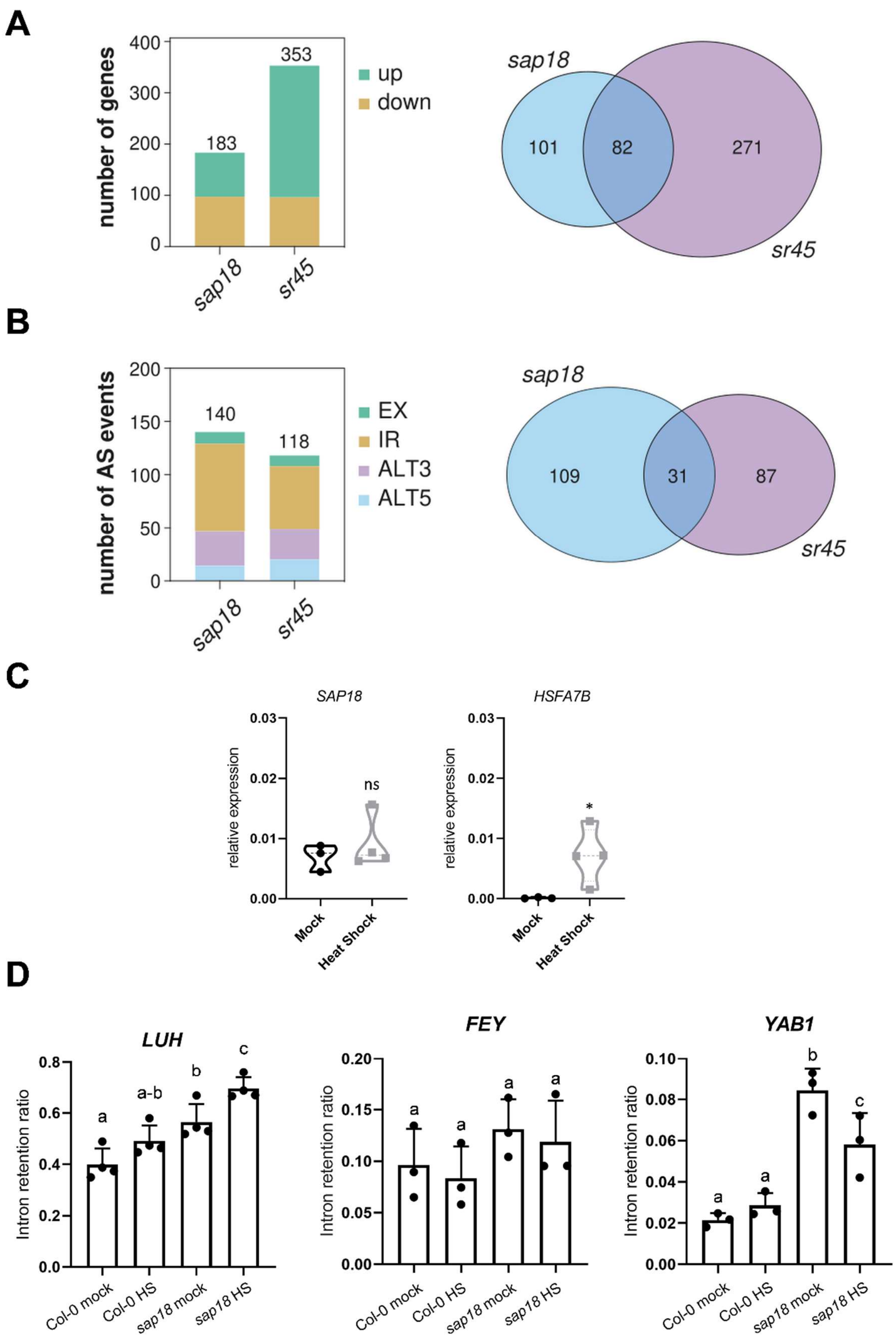
A
number of genes
400
300
200
100
0
183
353
up
down
sap18
sr45
sap18
101
82
271
sr45
B
number of AS events
200
150
100
50
0
140
118
EX
IR
ALT3
ALT5
sap18
sr45
sap18
109
31
87
sr45
C
SAP18
HSFA7B
relative expression
0.03
0.02
0.01
0.00
ns
*
Mock
Heat Shock
D
LUH
FEY
YAB1
Intron retention ratio
0.8
0.6
0.4
0.2
0.0
a
a-b
b
c
0.20
0.15
0.10
0.05
0.00
a
a
a
a
0.10
0.08
0.06
0.04
0.02
0.00
a
a
b
c
Col-0 mock
Col-0 HS
sap18 mock
sap18 HS

Supplementary Figure 8. (A) Diagrams showing differential expressed genes in *sap18* and *sr45* mutants 10-day-old seedlings in comparison with Col-0. (B) Diagrams showing alternative splicing in *sap18* and *sr45* mutants 10-day-old seedlings in comparison with Col-0. Full list of genes is presented in Supplementary Table 8. (C) Expression of *SAP18* transcript under heat stress conditions. *SAP18* mRNA transcript levels were measured under control (mock) and heat stress conditions (3h at 37°C). *ACTIN* was used as a normalization control for qRT-PCR analysis. Accumulation of the HS-inducible gene HSFA7B was included as positive control. Primers are listed in Supplementary Table 6. (D) Quantification of intron-retained isoforms in Col-0 and *sap18* mutant before and after a 3h heat shock (HS) treatment at 37 °C. Error bars show the standard deviation from the mean with 3 independent experiments for *FEY* and *YAB1* and 4 for *LUH*. Significance testing was performed using one-way ANOVA. ‘a’, ‘b’, and ‘c’ stand for groups presenting statistically significant differences.
