# Supplementary material for: Nucleo-cytoplasmic distribution of SAP18 reveals its dual function in splicing regulation and heat-stress response in Arabidopsis
Source: Plant Commun. 2024 Oct 31;6(1):101180. doi: 10.1016/j.xplc.2024.101180 (PMC11784288; doi:10.1016/j.xplc.2024.101180)
Supplement: Supplemental Table 6. Primers used in this study [file mmc7.pdf]

Name	Use	Sequence
*SAP18*_CDS_F	Cloning	CACCATGGCTGAAGCAGCGAGAAGACAAGGT
*SAP18*_CDS_nostop_R	Cloning	CTTGTAAATTGCCACATCCAGATAATCTC
*SR45*_CDS_F	Cloning	CACCATGGCGAAACCAAGTCGTGG
*SR45*_CDS_nostop_R	Cloning	AGTTTTACGAGGTGGAGGTG
*ACINUS*_CDS_F	Cloning	CACCATGTCGTCATCGCCTTTTCCAG
*ACINUS*_CDS_nostop_R	Cloning	CTTGTTATTATTCGCTGCAAGTTTAG
*MLP329*_CDS_F	Cloning	CACCATGGCGACATCGGGAACATA
*MLP329*_CDS_nostop_R	Cloning	GGCTTTGAGAACATGCTCGT
*DCP5*_CDS_F	Cloning	CACCATGGCGGCTGATAATACGGG
*DCP5*_CDS_nostop_R	Cloning	GGTAGTACGATTTGATACGC
*sap18*_salk_083076_F	Genotyping	GGCAAAGAAGATCGGGTAGTC
*sap18*_salk_083076_R	Genotyping	ACCACCACTCTGCAATCAAAC
*sr45*_salk_004132_F	Genotyping	TTTTGTTTTCCTTGTGTTGGC
*sr45*_salk_004132_R	Genotyping	GATTGGAGATCTTCTGGGAGG
*acinus*_salk_078554_F	Genotyping	CCCAAGAACCAGCAAGATCAC
*acinus*_salk_078554_R	Genotyping	ACCCACTACAACACCAAGGT
SALK Lbb1.3	Genotyping	ATTTTGCCGATTTCGGAAC
*YAB1*_F_IR	Intron retention rate	GAGGAGCTGAGAGATGCACCG
*YAB1*_R_IR	Intron retention rate	TTGATGAATCGGTTATATGCGGA
*YAB1*_F_IR2	qPCR	AAAGCACCACCCGTTAACCG
*YAB1*_R_IR2	qPCR	TGGCGATTCATCATCACAACCA
*LUH*_F_IR	Intron retention rate	TGATGGAGACGGAGGAAGCTT
*LUH*_R_IR	Intron retention rate	TATCATGTCCAGCGCTAGCCA
*FEY*_F_IR	Intron retention rate	ATGCATAGTGTCGGTTTTGTTGAC
*FEY*_R_IR	Intron retention rate	ACATTTGTTAGGACAACACCAGGG
*FEY*_F_IR2	qPCR	TGCCGTAGTTTTCATCTGCCG
*FEY*_R_IR2	qPCR	ACTCCTGTTTCCAGAGGAAGCT
*SAP18*_qF	qPCR	GAAGACAAGGTGGTGGGAGA
*SAP18*_qR	qPCR	GGTTCTTTGCCTCTCACAGC
*ACTIN*_qF	qPCR	CACTTGCACCAAGCAGCATGAAGA
*ACTIN*_qR	qPCR	AATGGAACCACCGATCCAGACACT
*HSFA7B*_qF	qPCR	CTATGGAGGGATTGCAGGAAG
*HSFA7B*_qR	qPCR	CACACGACAAAACTGATGCC

Supplementary Table 6. Primers used in this study.
