# Supplementary material for: Nucleo-cytoplasmic distribution of SAP18 reveals its dual function in splicing regulation and heat-stress response in Arabidopsis
Source: Plant Commun. 2024 Oct 31;6(1):101180. doi: 10.1016/j.xplc.2024.101180 (PMC11784288; doi:10.1016/j.xplc.2024.101180)
Supplement: Supplemental Table 7. DNA sequence of the fragments synthesized for cloning SAP18 genomic construct (pSAP18) — Sequences highlighted in gray correspond to UTRs. Sequences in bold correspond to adaptors used for Golden Gate cloning. Underlined ATG corresponds to the SAP18 start codon. [file mmc8.pdf]

Name	Type	Use	Sequence
SAP18_promoter+5' UTR	Fragment	**PU** Adaptors for Golden Gate cloning	**CACTCTGTGGTCTCAGGAG**atagaaaaatgaaagCTTATCAAaagttagtgggct<br>acatctacattatattaaaaaaatatatatatatatatatatatatatatatatatatatatatcaataacta<br>gatcggacagtgaccaaaCCAATCTCGTTGTGTCCAATCCAAGAAAATAACA<br>AAGGAAAGTCGTACGTTTGATGATATaaaaaCCtttttATGTTTGGACGAG<br>TTTCATAGAACATGGTGGACAAAATTATAGTACTATATACTATTTAGTA<br>TCTCCTTTTGaaaaaCAGTAAGCCATGTAAATGAATTTGTACAATTAAC<br>AGCTATTTaaaaaGCAaaaaaaaaaaCAGTGAATGTTTATTTACAAAGTTC<br>CGAAGGGAAGAGGTTGTGGTAGTGGTTAGGGGCATGCCAAATACGG<br>AAAGGGAGGCAAAGAAGATCGGGTAGTCTTCATAATTTCATACTTTTC<br>ACTTTGTAGTAATCTTTCCTTTTCttttttAATTTCATCACCAATGTTTCTTT<br>GTTCAAATTCATATCCACACACTCTTTTGGAAATGGCACTGCAGCTTC<br>ATGTGAGGTGTGGGGTCATTGCGCCTTATCGTCTGAAAATGTTACCC<br>TCTTGACATTCAATGACCCCATTTTCTTATTTTCCATAATTTCTTTTAC<br>CATTTTACTATAACAAGAATGATGCAAAAGGAATAGGCaaaaaaCAGCA<br>TATTAttttttAGTGGGATCCTAGTAGGTCACTTGAAGTCTCTATGCATTG<br>ATTATGGATTGCTATATTTGTCTCTCtttttAtttttGAATTTCGAGTTAGTGA<br>TCTTCAATGTGTGTTTCAAATATAGATATTCAAAGGTTATAAACCAAGA<br>AAAGaaaaaGTAGaaaaaaaaatatatatatTTCAAATCTTTTAAACGGACCATT<br>GGCAACAAAATCTATAAACGAGTTAGGCCCAAATTGTTGTACAAAAG<br>GCCCATTAGGTTAAATAAGTATGGGCCTAAAGCGGTCCGATCAATCT<br>CTGTGCCATTACCTTCTTCTTCGTCGTAGTGATTTCTCTCGAGAGaaa<br>aaGAGAG**AATGTGAGACCACGAAGTG**
SAP18_ORF	Fragment	**SC1** Adaptors for Golden Gate cloning (C-term tagging)	**CACTCTGTGGTCTCAAATG**<u>ATG</u>GCTGAAGCAGCGAGAAGACAAGGT<br>GGTGGAAGACCATTACCGCCACCTCCGAGAGGCGTTAACCAACAAC<br>CTCCTCGCCCTAAACCTGAACCTGTCGATCGTGAAAAGGTTTGTGTT<br>CGATTTCATTATTCGCGAATCGTTGAATCATCATCCTTTAGGGTTTCC<br>TTTTAGGGtttttATCTGTACTTATTGATTTCTTTATCTTTAACCTTCGAAT<br>CGCAGACATGTCCCCTTCTTCTTCGTGTTTTCACCAAGGTACTCTTCT<br>TCACTGATTTTACTGAATGCAAGTTTAATCACTGGAGAACTGGCTATG<br>TTTGTTTCTGAATTAGTCTGCAAATTAGGATTAATTGGGTAGGTTTGA<br>TTGCAGAGTGGTGGTCATCATACTAGTGAAGATTATGCTGTGAGAGG<br>CAAAGAACCAAAGGATGAAGTTCAAATCTACACTTGGAAAGATGCCA<br>GTCTTCGCGAGCTAACAGATTTGGTATGTCTTCTACCATTAAGATTTA<br>GATTGGGTGCTTGAGACAACAATAAAGCAGCTTGCTTTGAGAACTTT<br>TCTTGTGTAGTTTTGtttttACAAATATGACGGATTTTCAGCTAAAGGACT<br>CACTTTGtttttACTGCTGAGGAGTGCTGATTAAAATTCACAtttttgggggTTT<br>TGTGTAACGTACAAAGGTGATGCTTCTCTCATAAGCACAATGTTAAAT<br>CACGTTTTCATTGCAGGTTAAAGAAGTTTCTGTAGCAGCTAGGAGAA<br>GGAATGCTAGATTGTCTTTTGCGTTTGTTTATCCTAACAATAAAGGTG<br>GCTATAATGTGAGAGAGGTAAGATTACGTTCTCTTCACAATCTTGATC<br>AGATAAGTTGTCACCGTAGCCACGCATGATCATTGATGCATTCTAGA<br>GTTTCTGTGTTTTCCCCTGCCACACCTCTTTGTCGCACATGTAGCTAC<br>CAGTATCATTCGGAGTTTTGTCAACAATGTTTCTCTTGTACATGTCTTT<br>CGCTTATTGACAACTCATCTTCTCATGGTTATTGAtttttGAAACTTCAGA<br>AAGACCACATAATCATAGCTAGTTACTACAGAATGAAGTAATTTGATT<br>GAATGGATGCTTTCAGATTCAATCGTTATGGCTTTACTTGTTCTCTTG<br>TGATGTTTGCATATGTATTCAAGAACCCATGAGAAGAGTCTGATCTTG<br>TGGTGTTGTTTCTGTTAATTGATAAAGGTTGGGGAGACGATGGCTTA<br>TCCAAACCGAAAACAACCAGATGACAGTAAAACGCTTTCCGAACTTC<br>CGTTTGAGGTAAGttttttACATGCATAGAAGATCATCTTGTATGATGATT<br>ATATAGTCTATAACATTAAtttttCATATTCTGTTACCCAAGTAGCTATAAC<br>TGAAGTTGATCAATCTATCTGCAGATTGGAGATTATCTGGATGTGGC<br>AATTTAC**GGTGTGAGACCACGAAGTG**
SAP18_3'UTR+Term	Fragment	**T** Adaptors for Golden Gate cloning	**CACTCTGTGGTCTCAGCTT**GAGGTTTCTGTTTCTTGTTAAGTGGGATT<br>TGGGTGCGTGCACAATGGAAGAAGTACACATTCATATGGTTGTATCA<br>TCTTAAACTTCGTTTAGACATGTTTGTATTTTTTGGATCTAGTCTATGT<br>ACTAATGTCTAATGGTTTAGTATGTTATCCTGCTTTTTTTGTTTAACGT<br>GTTTTCTTTTCTAAAGAACCAAAAAAAAAGATGGAAGAACTCAAGCCAT<br>TTTATATACAAAAAGGTATAAATTTAGACTATCTATAGACTATAGCACA<br>TGAATATGTATTAAATTAAGATTTCAACCCAATTTACTTTTATTAGTAG<br>AATTAATACTCGCACTTAACGTCGGAGAAGTAGCTGAGaaaaaGAAAT<br>CGTGaaaaaaaTGAGAGAAGAGGATGAATTTAGACAACATTAATCATCA<br>AAACTATAGTGATAAACATAGAAGAGTGGGCAATGCATTTCAAATATT<br>CTCaaaaaTAAAGATATAGTTATGGAATTATACAAAAGTTTAATAGCAA<br>AATATGAACGTTGCGTTTACGATTCCCTATATTGTTCCAACGTTAAGT<br>TTGATTAGACTTGAAAGTTGAAAGTATAATAAAATTTAAACGTTGAAAC<br>GGTAACATTTTATTACCGTTAGAAGATAATATTTTATTACaaaaaTTCAG<br>ATAAATGGCTCTCTATAAATACTCCCATCTTGGGTCAGAAATCGGAAT<br>CCTCTTCATTGGTTCATATTTTACAAAATGTCGAGAAACAATCTGtttttA<br>TTTCTTAATTCTGGTTCTTCCACTTGAGTCAAGACCTCATTCAAACAA<br>ACATAAAGCATCATCCTTACAAGGTAAAATACTCTGGTCTAAATCCTC<br>TAGTGTTTACCGTTAAATGGTttt**CGCTTGAGACCACGAAGTG**

Supplementary Table 7. DNA sequence of the fragments synthesized for cloning SAP18 genomic construct (pSAP18). Sequences highlighted in grey correspond to UTRs. Sequences in bold correspond to adaptors used for Golden Gate cloning. Underlined ATG corresponds to SAP18 start codon.
